# A new type of DNA phosphorothioation-based antiviral system in archaea

**DOI:** 10.1038/s41467-019-09390-9

**Published:** 2019-04-11

**Authors:** Lei Xiong, Siyi Liu, Si Chen, Yao Xiao, Bochen Zhu, Yali Gao, Yujing Zhang, Beibei Chen, Jie Luo, Zixin Deng, Xiangdong Chen, Lianrong Wang, Shi Chen

**Affiliations:** 1 Key Laboratory of Combinatorial Biosynthesis and Drug Discovery Ministry of Education, School of Pharmaceutical Sciences, Wuhan University, 430071 Wuhan, China; 2 Taihe Hospital, Hubei University of Medicine, 442000 Shiyan, Hubei China; 3 Brain Center, Zhongnan Hospital, Wuhan University, 430071 Wuhan, China; 4 School of Biology and Pharmaceutical Engineering, Wuhan Polytechnic University, 430023 Wuhan, China; 5 College of Life Sciences, Wuhan University, 430071 Wuhan, China

## Abstract

Archaea and Bacteria have evolved different defence strategies that target virtually all steps of the viral life cycle. The diversified virion morphotypes and genome contents of archaeal viruses result in a highly complex array of archaea-virus interactions. However, our understanding of archaeal antiviral activities lags far behind our knowledges of those in bacteria. Here we report a new archaeal defence system that involves DndCDEA-specific DNA phosphorothioate (PT) modification and the PbeABCD-mediated halt of virus propagation via inhibition of DNA replication. In contrast to the breakage of invasive DNA by DndFGH in bacteria, DndCDEA-PbeABCD does not degrade or cleave viral DNA. The PbeABCD-mediated PT defence system is widespread and exhibits extensive interdomain and intradomain gene transfer events. Our results suggest that DndCDEA-PbeABCD is a new type of PT-based virus resistance system, expanding the known arsenal of defence systems as well as our understanding of host-virus interactions.

## Abstract (web summary)

The prokaryote defence system Dnd relies on phosphorothioation (PT) of DNA backbone to distinguish between self and non-self DNA. Here, Xiong et al. describe a previously uncharacterized PT-based antiviral system that is independent of the canonical Dnd and is widespread in Archaea and Bacteria.

## Introduction

Viruses are the most abundant biological entities in the biosphere and are estimated to outnumber their bacterial and archaeal hosts by tenfold. The constant threat of virus predation has led to the evolution of diverse defence mechanisms that target nearly every stage of the viral infectious cycle. These mechanisms involve inhibiting adsorption, blocking viral DNA injection, restriction-modification (R-M), limiting phage growth, toxin-antitoxin systems, abortive infection and CRISPR/Cas systems, as well as the newly discovered prokaryotic Argonaute, BREX, DISARM, Zorya and Dnd defence systems^1–6^.

Among these mechanisms, the use of R-M systems is the most prevalent and best-characterised antiviral approach among Bacteria and Archaea. Generally, R-M systems consist of two contrasting enzymatic activities: (1) a methyltransferase (MTase) that catalyses the transfer of a methyl group to DNA nucleobases within a particular sequence motif of ‘self’ DNA and (2) a restriction endonuclease (REase) cognate that identifies and destroys invading foreign DNA that harbours the same DNA motif when not methylated. Four major types of R-M systems (I, II, III and IV) are classified based on their subunit composition, cofactor requirements, sequence recognition and cleavage mechanisms^7^. Type I R-M systems comprise three subunits, R (restriction), M (modification) and S (specificity), and the resulting complex binds to a bipartite sequence and requires ATP hydrolysis to cleave at a distant site following DNA translocations^8^. Type II systems are the simplest, which contain separate MTase and REase enzymes to act independently. Type III systems are multifunctional oligomeric proteins consisting of a Mod and a Res subunit. The Mod subunit alone can function independently as a MTase to recognise non-palindromic sequences, only one strand of which can be methylated, whereas the Res subunit needs to pair with the Mod subunit for restriction activity^9^. Type IV systems have no methylase activity and type IV REases recognise and cleave only modified DNA^10–12^. Although type I–III R-M systems are tremendously diverse, the associated DNA modifications most commonly occur in the form of methylation of nucleobase moieties, such as N^6^-methyl-adenine, N^4^-methyl-cytosine and C^5^-methyl-cytosine.

The Dnd defence system has recently been recognised as a new component of the bacterial innate immune system, in which phosphorothioate (PT) modification is used as a marker to distinguish between self and non-self DNA, analogous to methylation-based R-M systems^13,14^. In contrast to DNA methylation at nucleobases, PT modification occurs on the DNA sugar-phosphate backbone, where replacement of the non-bridging oxygen by sulphur confers nuclease resistance at the PT linkage^13,15^. The Dnd system is organised into three parts: (1) DndACDE proteins form a complex and act as the modification component to catalyse DNA PT modification in a sequence-selective manner^16,17^; (2) DndB is a transcriptional repressor capable of regulating expression of the *dndBCDE* cluster and the resulting PT level^18^ and (3) DndFGH proteins function as the R component to recognise and destroy non-PT-modified foreign DNA^14,19^. All three *dndFGH* genes are essential to distinguish and restrict the transformation of non-PT-modified incoming plasmid DNA in *Salmonella enterica*^4^. Once PT modification is abolished, *S. enterica* suffers double-stranded DNA damage from the unrestrained restriction activity of DndFGH, which consequently leads to growth defects and triggers the SOS response^14,20^. Although the enzymatic activities and mechanism of action of DndFGH is currently unclear, these features resemble the well-characterised ‘self/non-self discrimination’ strategy of methylation-based R-M systems.

To prevent REase attack on resident DNA, the recognition motifs are generally nearly completely methylated by the MTase counterpart. However, this situation is not observed for Dnd defence systems. Indeed, only 4855 of 40,701 complementary 5ʹ-GAAC-3ʹ/5ʹ-GTTC-3ʹ consensus sequences found in the *Escherichia coli* B7A genome are PT modified to 5ʹ-G_PS_AAC-3ʹ/5ʹ-G_PS_TTC-3ʹ (PS: phosphate-sulphur linkage), even in the presence of active DndFGH, ruling out the known R-M mechanisms.

Although the number of described archaeal viruses accounts for only 1% of all reported prokaryotic viruses, the archaeal viruses exhibit enormously diversified morphotypes and genome contents^21–24^. Many of the unique morphologies of archaeal viruses, including coil-shaped, bottle-shaped, spherical and droplet-shaped with beard-like fibres, have not been observed among bacterial or eukaryotic viruses^21^. Consistent with the uniqueness of viral morphotypes, the genome contents of these archaeal viruses are distinctive, with ~75% of genes encoding functionally unknown proteins^25^. Although the current knowledge of archaeal viruses has merely touched the tip of the iceberg, one may imagine the development of diverse defence systems during the coevolutionary arms race between archaea and viruses given the distinct morphological and genomic properties of viruses.

Indeed, as in Bacteria, an astonishing array of defence systems have developed among Archaea to restrict invasive viruses and plasmids. For example, 96% of the sequenced bacterial genomes harbour R-M systems, while 384 (98%) of the 390 currently available archaeal genomes encode R-M systems (http://rebase.neb.com/rebase/rebase.html)^26^, as of the time of writing of this manuscript. However, only a few archaeal R-M systems have been investigated to date, including R.PabI from the hyperthermophilic *Pyrococcus abyssi*, R.PspGI from *Pyrococcus* sp. strain GI-H, R.SuaI from the thermoacidophilic *Sulfolobus acidocaldarius* and their cognate MTases^27–30^. Moreover, archaea wrap their DNA into histone-DNA complexes with the same geometry as DNA in the eukaryotic nucleosomes^31^, prompting us to investigate the aspects of PT modification and PT-related functions in Archaea.

In this study, we first identify PT modifications in halophilic and methanogenic archaea that show diverse sequence specificities and abundance comparable to those observed in bacteria. However, no trace of the restriction component DndFGH is detected in any of the archaeal strains assessed. Instead, we report that PT-modification genes, such as *dndCDEA* in the halophilic archaeon *Haloterrigena jeotgali* A29, can defend against viral attack together with *pbeABCD*, a conserved 4-gene cassette sharing no sequence homology with DndFGH. Moreover, the defence mechanism involves the DndCDEA-mediated PT modification of the self-DNA sequence and the use of PbeABCD to halt virus propagation by inhibiting DNA replication in a PT-dependent manner. Unlike the DndFGH-mediated defence mechanism, DndCDEA-PbeABCD does not function through the degradation or cleavage of viral DNA, highlighting a novel mode of action that is distinct from R-M activity and expanding the arsenal of known defence systems, as well as our understanding of host–virus interactions.

## Results

### Occurrence of DNA PT modifications in Archaea

**Fig. 1:**
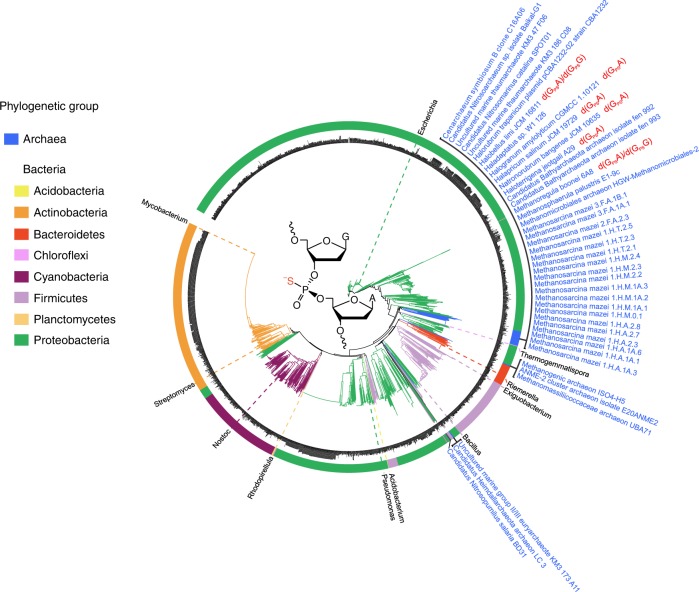
Maximum likelihood phylogeny of 2642 homologues of the *H. jeotgali* A29 DndCD identified in Archaea and Bacteria. The strain names of 42 archaea, as well as the phosphorothioate (PT) sequence contexts identified in this study are displayed as noted. Each prokaryotic phylum is represented in a distinct colour (see the legend). Bacterial strain names are also provided in Supplementary Data 1. For clarity, at least one familiar genus per colour segment were labelled. The structure of PT-modified *d*(G_PS_A) in the *R*_P_ configuration is shown in the centre of the tree. Source data are provided as a Source Data file

Although our understanding of *dnd* systems and PT modifications in Bacteria has dramatically expanded in the past decade, PT modification and its physiological relevance have not been characterised in Archaea, which comprise up to 20% of the total biomass on Earth^21^. In this study, we first identified DNA PT modifications in five archaea with homologous PT-modification genes. Based on mass spectral analysis, nuclease-resistant PT-linked dinucleotides were produced upon enzymatic hydrolysis of archaeal DNA (Fig. 1 and Supplementary Fig. 1). The following strains were observed to harbour PT-modified *d*(G_PS_A): three halophilic archaeal strains, *H. jeotgali* A29, isolated from salt-fermented shrimp jeotgal, and *Halapricum salinum* JCM 19729 and *Halobellus limi* JCM 16811, isolated from solar salterns in Korea and China, respectively; and one haloalkaliphilic archaeal strain, *Natronorubrum bangense* JCM 10635, isolated from a soda lake in Tibet. *H. limi* JCM 16811 also possesses a detectable level of *d*(G_PS_G). An acidophilic methanogen, *Methanoregula boonei* 6A8, isolated from an acid peat bog in the United States, also shows PT modification at *d*(G_PS_A) and *d*(G_PS_G) of the same order of magnitude (Table 1). These findings reveal the diversity of PT consensus sequences among Archaea. Next, we determined that the *dndCDEA* operon of *H. jeotgali* A29 was capable of conferring *d*(G_PS_A) modification upon transfer to a new host, *Natrinema* sp. CJ7-F, an extreme haloarchaeon without endogenous *dnd* genes (Supplementary Fig. 1).

**Table 1:** DNA phosphorothioate (PT) modifications in archaeal strains

Strains	GenBank accession no.	*d*(G_PS_A)	*d*(G_PS_G)	Total PT
		(per 10^4^ nt)		
*H. jeotgali* A29	CP031298–CP031304	2.8 ± 0	NA	2.8 ± 0
*N. bangense* JCM 10635	CP031305–CP031309	1.8 ± 0.1	NA	1.8 ± 0.1
*H. limi* JCM 16811	CP031311–CP031314	3.0 ± 0.3	^a^	3.0 ± 0.3
*H. salinum* JCM 19729	CP031310	1.8 ± 0.1	NA	1.8 ± 0.1
*M. boonei* 6A8	CP000780	1.4 ± 0.5	2.1 ± 0.7	3.4 ± 1.2

Values represent the mean ± SD of three analyses of 10 μg of archaeal DNA. Source data are provided as a Source Data file

NA, not applicable

^a^Indicates that the PT-linked dinucleotides were detected but were below the limit of quantification

We next used the DndCD protein sequences (DndE is too small to be annotated in some genomes) of *H. jeotgali* A29 as a query to explore the occurrence and diversity of Dnd systems across archaeal genomes. Using BLAST, we searched the homologues of DndCD in the NCBI databases of non-redundant nucleotide collections (nt), RefSeq Genome (refseq_genomes), whole-genome shotgun contigs (WGS), high-throughput genomic sequences (HTGS), reference genomic sequences (refseq_genomic), and genomic survey sequences (GSS). We filtered the results based on an *e*-value of ≤ 10^−10^ and discarded BLAST hits with aligned lengths of ≤ 30% of the query proteins. A total of 2642 occurrences of *dndCD* were identified nested within 2600 bacterial and 42 archaeal genomes (Fig. 1 and Supplementary Data 1). The 42 Archaea harbouring DndCD homologues span the phylogenetic diversity of archaeal lineages, including Methanomicrobia (23), Haloarchaea (7), Thaumarchaeota (6), Bathyarchaeota (2), Thermoplasmata (2), and Heimdallarchaeota (1), as well as an uncultured marine group II/III euryarchaeote KM3_173_A11 (Supplementary Data 1). According to the phylogenetic tree of the 2642 DndCD proteins (archaeal and bacterial DndCD proteins), the archaeal strains do not phylogenetically cluster together but instead are grouped with bacteria, suggesting potential interdomain HGT of Dnd systems between Archaea and Bacteria (Fig. 1).

### Genome-wide PT features in Archaea

In contrast to the well-characterised *dndBCDE* cluster in bacterial genomes, *dndCDEA* clusters in *H. jeotgali* A29, *N. bangense* JCM 10635, *H. limi* JCM 16811 and *H. salinum* JCM 19729 are not accompanied by *dndB*, which encodes a negative regulator that binds to the promoter region of the *dndBCDE* operon. Upon relief of DndB repression, the transcription of *dndBCDE* and the resulting PT frequency increased by 15- and 2-fold, respectively, in the bacterial strain *S. enterica* serovar Cerro 87^18^. However, in the four haloarchaeal strains, we observed *d*(G_PS_A) at an average frequency of ~2–3 PT per 10^4 ^nt, which is comparable to the frequency of ~3–8 PT per 10^4^ nt detected in bacteria^16^, prompting us to examine the PT features across the archaeal genomes (Table 1).

**Fig. 2:**
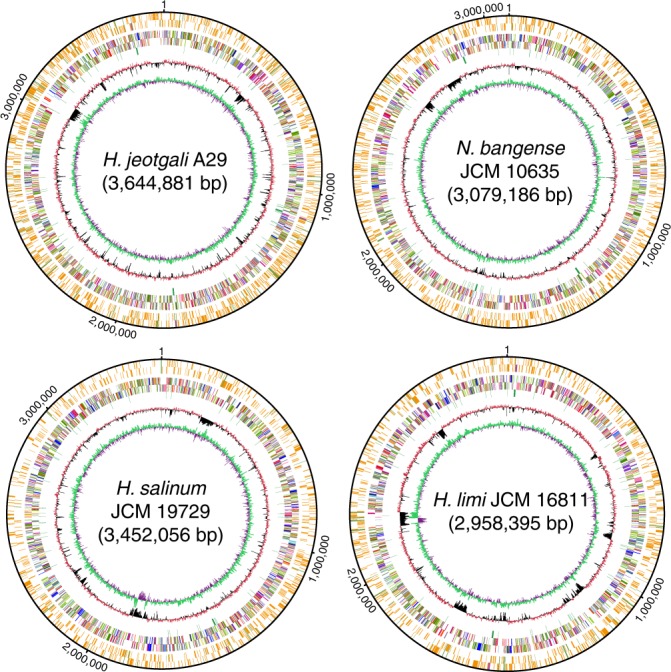
Phosphorothioate (PT) mapping across the genomes of *H. jeotgali* A29, *N. bangense* JCM 10635, *H. salinum* JCM 19729 and *H. limi* JCM 16811. From the outer to the inner circles: circles 1 and 2 (forward and reverse strands), PT sites in ORFs (orange), noncoding RNA (purple), and nonencoding regions (green); circles 3 and 4, predicted protein-coding sequences coloured according to COG functional categories; circle 5, tRNA/rRNA operons; circle 6, guanine-cytosine content; and circle 7, guanine-cytosine skew. Source data are provided as a Source Data file

To this end, we exploited the single-molecule real-time (SMRT) sequencing platform to profile the genome-wide PT sites^32,33^. The SMRT sequencing data immediately revealed the occurrence of *d*(G_PS_A) in a 4-bp consensus sequence of 5ʹ-G_PS_ATC-3ʹ in all four haloarchaea genomes (Supplementary Fig. 2). Only 2.6% (2451) out of 94,316, 2.1% (1614) out of 75,884, 2.4% (1442) out of 60,776, and 1.4% (895) out of 64,824 5ʹ-GATC-3ʹ sites in *H. jeotgali* A29, *N. bangense* JCM 10635, *H. limi* JCM 16811 and *H. salinum* JCM 19729 were detected to be PT modified, respectively (Supplementary Data 2). Although no further sequence constraint beyond the 4-bp 5ʹ-G_PS_ATC-3ʹ was identified, we observed sequence biases for PT modification-proximate bases, with a noticeable preference for 5ʹ-tG_PS_ATCc-3ʹ but an underrepresentation at 5ʹ-gG_PS_ATC(g/t)-3ʹ (Supplementary Table 1). No PT enrichment in particular DNA regions was observed across all four archaeal genomes (Fig. 2 and Supplementary Data 2). For instance, 1229 and 1222 5ʹ-G_PS_ATC-3ʹ sites were observed to be located on the (+) and (−) strands of the *H. jeotgali* A29 genome, respectively, with various spacing ranging from 4 to 36 kb. In addition, 915 out of 4745 open reading frames, 8 out of 13 rRNA genes and 31 out of 200 pseudogenes in *H. jeotgali* A29 harbour at least one PT site (Fig. 2 and Supplementary Data 2). While 1310 of 2451, 674 of 1614, 736 of 1442, and 244 of 895 genomic PTs were detected to be fully PT-modified (on both strands) 5ʹ-G_PS_ATC-3ʹ/5ʹ-G_PS_ATC-3ʹ in *H. jeotgali* A29, *N. bangense* JCM 10635, *H. limi* JCM 16811 and *H. salinum* JCM 19729, respectively, up to 47% (1141), 58% (940), 49% (706), and 73% (651) of PT sites occur in the form of hemimodified 5ʹ-G_PS_ATC-3ʹ/5ʹ-GATC-3ʹ (Supplementary Fig. 2). These results are in sharp contrast to the predominant full PT modification found in 5ʹ-G_PS_AAC-3ʹ/5ʹ-G_PS_TTC-3 in *E. coli* B7A^32^ and 5ʹ-G_PS_GCC-3ʹ/5ʹ-G_PS_GCC-3ʹ in *Pseudomonas fluorescens* pf0–1^34^, suggesting different PT physiologies among Archaea. Additionally, it was interesting to observe the presence of DNA methylation in a variety of sequence contexts, including 5ʹ-TCCGA^6m^A-3ʹ, 5ʹ-GAGG^6m^AG-3ʹ, 5ʹ-C^6m^ATTC-3ʹ, 5ʹ-CAG^6m^ATG-3ʹ, and 5ʹ-CG^6m^ATCC-3ʹ, by SMRT sequencing (Supplementary Table 2), which indicated their potential involvement in methylation-based R-M defence systems, epigenetic regulation, or other biological functions in Archaea.

### DndCDEA constitutes a novel antiviral system with PbeABCD

**Fig. 3:**
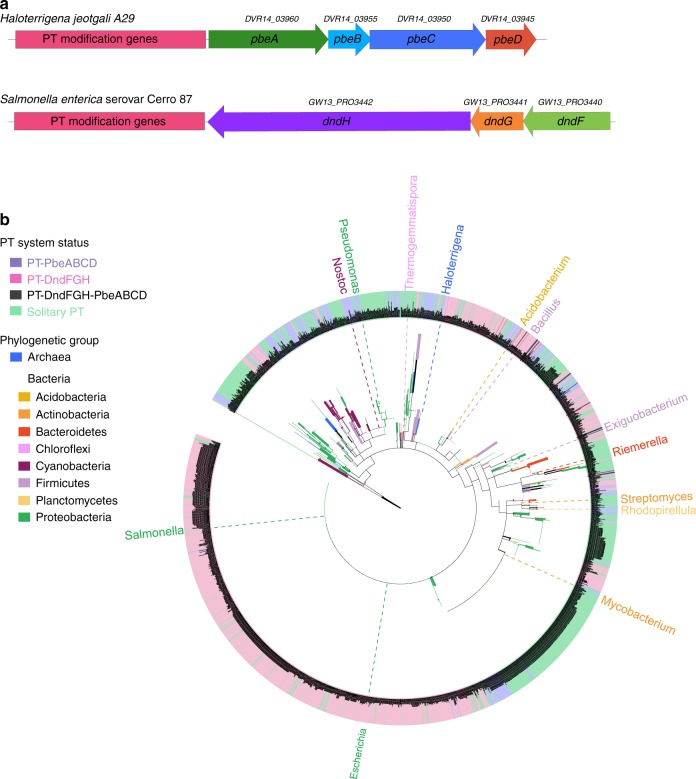
The diversity of phosphorothioate (PT) systems in prokaryotes. **a** The genetic organisation and gene orders of the PT-based PbeABCD and DndFGH systems are displayed. GenBank accession numbers for *pbeABCD* in *H. jeotgali* A29 are indicated. **b** The phylogenetic tree of 2361 archaeal and bacterial DndCD homologues is colour-coded by phylogenetic group (see the legend). Strains shaded violet, pink, black and green have DndCD homologues associated with PbeABCD, DndFGH, DndFGH-PbeABCD, or none, respectively. The widths of tree branches are proportional to their Bootstrap scores. For clarity, at least one familiar genus per colour segment were labelled. Source data are provided as a Source Data file

Based on these PT characteristics in Archaea, we sought to explore the genomic neighbourhoods of *dndCD* by selecting 2361 out of 2642 archaeal and bacterial *dndCD* with available flanking sequence data. The restriction component *dndFGH* was observed to be in close proximity (1 bp-20 kb) with 48.8% (1132) of the 2322 bacterial *dndCD* regions, agreeing well with our previous observations^34^. Conversely, no trace of *dndFGH* was observed within the vicinity of all 39 archaeal *dndCD*. Instead, the majority of these archaeal *dndCD* clusters are followed by a four-gene cassette arranged in a highly conserved order (Fig. 3).

Using the gene cassette consisting of *DVR14_03960*, *DVR14_03955*, *DVR14_03950* and *DVR14_03945* in *H. jeotgali* A29 as an example, the four genes are co-expressed as a single transcriptional unit, consistent with the idea that they are functional related (Supplementary Fig. 3). DVR14_03960 comprises three domains: a phospholipase D (PLD)-like domain (pfam13091), a res subunit of Type III REase (pfam04851) and a DEAD (Asp-Glu-Ala-Asp)-family helicase C-terminal (pfam00271) domain (Supplementary Fig. 4). The PLD domain has been identified as a component of a large superfamily of proteins associated with the hydrolysis of phosphodiester bonds, such as phospholipases, kinases, toxins and REases^35^. The genes *DVR14_03955* and *DVR14_03945* encode proteins of unknown function with only 66 and 152 aa in size, respectively. DVR14_03950 contains an N-terminal AAA domain (ATPases associated with diverse cellular activities; pfam13476), a domain that is present in a wide variety of proteins that often direct molecular remodelling events in an ATP-driven process^36^. Given the positioning of these genes relative to *dndCDEA* and the predicted nuclease-related functions of their encoded proteins, we speculated that this four-gene cluster may constitute a new defence system involving DNA PT modification in Archaea, and we named the four genes *pbeABCD* (for phosphorothioate-blocked DNA exclusion).

**Fig. 4:**
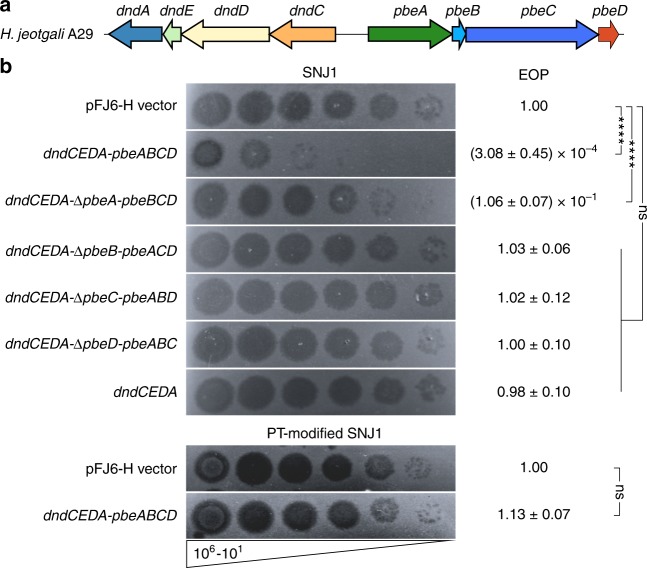
DndCDEA-PbeABCD confers protection against the haloarchaeal virus SNJ1. **a** The *dndCDEA-pbeABCD* locus of *H. jeotgali* A29 is displayed. **b** Five microlitres each of a tenfold serial dilution (10^6^–10^1^) of SNJ1 virus was spotted onto CJ7-F cells carrying pWHU3808-derived plasmids and expressing the genes indicated on the left. The EOPs are listed on the right. The EOPs were determined by dividing the SNJ1 titre obtained by plating on CJ7-F expressing *dndCDEA-pbeABCD* and derivatives by the titre of SNJ1 plated on CJ7-F(pFJ6-H). The values represent the mean ± SD for three independent experiments. ****p-value < 0.0001, using one-sample *t*-test (Hypothesis Testing). Ns, not significant. Compared to the strong protection against non-PT SNJ1, CJ7-F cells are susceptible to PT-modified SNJ1, even in the presence of DndCDEA-PbeABCD. Source data are provided as a Source Data file

To explore the involvement of *pbeABCD* in archaeal host–virus interactions, we cloned the *dndCDEA-pbeABCD* fragment from *H. jeotgali* A29 into the shuttle vector pFJ6-H, generating pWHU3808, followed by transformation into *Natrinema* sp. CJ7-F, a *pyrF*-deletion and plasmid-free derivative of *Natrinema* sp. J7-1 (Supplementary Table 3). We next challenged CJ7-F cells expressing *dndCDEA-pbeABCD* with the halophilic temperate sphaerolipovirus SNJ1. Using a double agar overlay plaque assay, we showed that *dndCDEA-pbeABCD* provided protection against SNJ1: the ability of SNJ1 to induce plaques was reduced ~10^4^-fold (Fig. 4).

To further examine the components that are essential for this antiviral activity, we constructed a series of pWHU3808-derived plasmids (pWHU3813~pWHU3816), each expressing DNA PT modification, as well as the individual in-frame deleted genes of the *pbeABCD* cassette (Supplementary Table 3). Deletion of the individual *pbeABCD* genes impaired protection against SNJ1, indicating that all four *pbe* genes are indispensable for mediating antiviral activity (Fig. 4). It is noteworthy that the contributions of four *pbe* genes against SNJ1 are not equal. To quantify the protection, we measured the efficiency of plating (EOP) of SNJ1 applied to CJ7-F cells expressing different parts of the *pbeABCD* cassette. In contrast to the absolute necessity of *pbeB*, *pbeC* and *pbeD*, CJ7-F cells expressing *dndCDEA-ΔpbeA-pbeBCD* still exhibited a low-level of resistance, although less than one order of magnitude, against SNJ1, suggesting that *pbeA* is partially required for defence (Fig. 4). To assess whether PT modification is sufficient to protect viruses against PbeABCD interference, we propagated SNJ1 in CJ7-F cells expressing only *dndCDEA*, yielding PT-modified SNJ1 (Supplementary Fig. 5). In sharp contrast to the strong protection observed against the non-PT-modified SNJ1 virus, CJ7-F cells expressing *dndCDEA*-*pbeABCD* displayed the same susceptibility to PT-modified SNJ1 as CJ7-F cells lacking *dndCDEA*-*pbeABCD* (Fig. 4).

It is, therefore, likely that *dndCDEA-pbeABCD* encodes R-M activity for which DNA PT modification serves as a recognition tag, allowing discrimination between self and non-self DNA. If the Pbe moiety is capable of independent action, then deletion of PT modification genes would likely be detrimental to archaeal cells because genomic 5ʹ-GATC-3ʹ sites would no longer be protected by PT from the restriction activity of PbeABCD. Surprisingly, it was easy to prepare the plasmids pWHU3809, pWHU3810, pWHU3811 and pWHU3812 harbouring individually in-frame-deleted *dndCDEA* genes but a complete *pbeABCD* (Supplementary Table 3). Indeed, these plasmids exerted no notable toxicity towards CJ7-F cells and failed to reduce the ability of SNJ1 virus to cause plaque formation, despite the expression of *pbeABCD* (Supplementary Fig. 6).

Thus, it is possible that the antiviral activity of PbeABCD depends on DNA PT modification in *trans* or on DndCEDA proteins. To address this possibility, we attempted to construct a plasmid (pWHU3789) expressing DndCDEA_C344S_-PbeABCD with a single cysteine replaced by serine in DndA. The catalytic cysteine of DndA (C344), corresponding to C327 in *S. lividans* DndA, is responsible for the nucleophilic attack on the substrate cysteine^37^. Thus, although this mutation would abolish DNA PT modification at 5ʹ-G_PS_ATC-3ʹ, the integrity of the DndCDEA assembly would be retained. The immediate observation was that in spite of the deficiency of PT modification, *pbeABCD* was still transcribed in CJ7-F(*dndCDEA*_*C344S*_, *pbeABCD*), excluding the possibility that DndA or PT acts as a transcriptional regulator of the *pbe* operon (Supplementary Fig. 7a, b). However, while the transformation of CJ7-F hosts with the pWHU3789 plasmid occurred at normal frequency, the CJ7-F(pWHU3879) transformant restored the sensitivity to SNJ1 (Supplementary Fig. 7c). This result suggested that PbeABCD exerted antiviral activity depending on the presence of PT modifications rather than protein–protein interactions with DndCDEA. This activity differentiates the PbeABCD-mediated defence mechanism from that of DndFGH because the latter still exerted a bactericidal effect, even in the absence of PT modification.

### PbeABCD allows virus adsorption but inhibits DNA replication

**Fig. 5:**
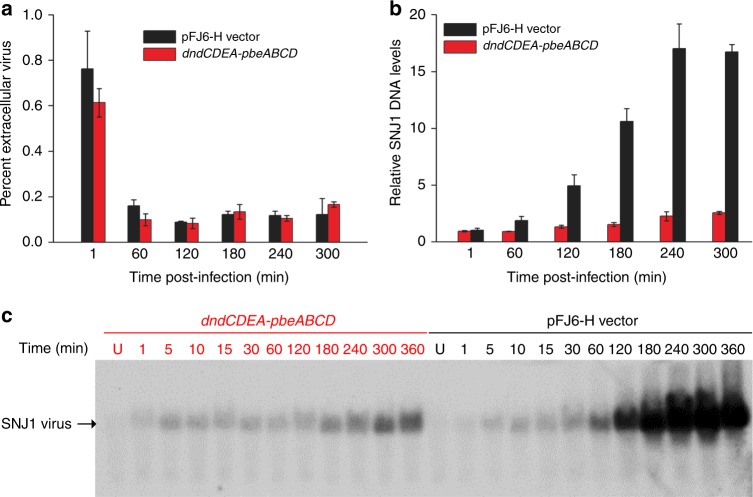
Adsorption and DNA replication of the SNJ1 virus in CJ7-F cells expressing or lacking DndCDEA-PbeABCD. **a** Adsorption of SNJ1 to *dndCDEA-pbeABCD*-containing CJ7-F cells (red) is not impaired compared to that observed for CJ7-F cells containing the empty vector pFJ6-H (black). After the infection of logarithmic-stage cultures (OD600 = 0.3) with SNJ1 at an MOI = 1, samples were collected at 60-min intervals, and the extracellular (unabsorbed) viral concentration was measured and compared to the initial viral concentrations. The bars represent the means of three experiments, and the error bars represent the SD of the mean. **b** Real-time qPCR analysis to determine the replication efficiency of the virus SNJ1 in CJ7-F cells expressing (red) or lacking (black) *dndCDEA-pbeABCD*. Primers amplifying *repA* and *radA* were used to determine the concentration of SNJ1 DNA and CJ7-F chromosomes, respectively. The 2^C^_T_^(radA) ^– ^C^_T_^(repA)^ method was used to determine the relative levels of SNJ1 DNA in CJ7-F hosts. **c** Southern blot analysis of the SNJ1 genome during the infection cycle. Numbers indicate the time (min) following infection. A probe was designed corresponding to positions 9243–9732 nt in the SNJ1 genome. Source data are provided as a Source Data file

To gain further insight into the mechanism of action of the PbeABCD-mediated PT defence system, we examined whether the system provides protection by hindering virus attachment. To this end, we compared the efficiency of SNJ1 adsorption to *dndCDEA-pbeABCD*-containing and *dndCDEA-pbeABCD*-lacking CJ7-F cells. The result showed that SNJ1 viruses were able to adsorb to the archaeal cells at the same rates regardless of the presence of the *dndCDEA-pbeABCD* system (Fig. 5a). This finding suggests that DndCDEA-PbeABCD does not offer protection by interfering with virus attachment.

We next monitored the replication efficiency of SNJ1 viral DNA during the time course of the infection using real-time quantitative polymerase chain reaction (qPCR). The PCR products for *repA*, which is located within the replication region of SNJ1, and *radA*, a single-copy gene located on the CJ7-F chromosome, were used to estimate the relative viral DNA concentrations compared to archaeal genome equivalents^38^. After 5 h of infection, a 2.5-fold increase in the intracellular SNJ1 genome level was observed in CJ7-F (*dndCDEA*, *pbeABCD*), whereas a 16.7-fold increase was detected in CJ7-F containing the empty vector (Fig. 5b). These results revealed that SNJ1 DNA can be successfully injected into *Natrinema* cells but that the subsequent viral genome replication process is significantly inhibited by the DndCDEA-PbeABCD system. This observation, along with the results of the adsorption assays, indicated that the replication inhibition likely results from viral DNA cleavage or degradation.

To examine the integrity of SNJ1 DNA, we performed a Southern blot analysis using total DNA isolated from CJ7-F cells treated with SNJ1 at various time points after infection. With increasing infection time, extensive SNJ1 DNA accumulation was observed in *dndCDEA*-*pbeABCD*-lacking but not *dndCDEA*-*pbeABCD*-containing CJ7-F cells, indicating that DndCDEA-PbeABCD halts viral infection at an early stage (Fig. 5c). Notably, SNJ1 DNA remained intact with no sign of cleavage or processive degradation, even 6 h after infection of CJ7-F cells expressing the DndCDEA-PbeABCD system (Fig. 5c). Consistent with the real-time qPCR results, the Southern blot analysis showed a marginal increase in SNJ1 DNA with an extended infection time, even in the presence of DndCDEA-PbeABCD. Considering the self/non-self discrimination strategy, one explanation for this result is that once SNJ1 DNA acquires PT modification prior to the action of PbeABCD, it is regarded as self-DNA and can replicate in CJ7-F cells, highlighting an evolutionary arms race between the PbeABCD-mediated defence barrier and parasitic DNA. Collectively, these results suggested that DndCDEA-PbeABCD confers resistance against invading viruses via a mechanism other than the direct cleavage or degradation of viral DNA used in canonical R-M systems.

### PT-based PbeABCD systems are widespread in microbial genomes

After discovering this new PT-based defence activity in Archaea, we re-examined *pbeABCD*-adjacent genes among the 2361 prokaryotic *dndCD* regions. As the gene products of *pbeB* and *pbeD* are too small to be annotated in some genomes, we used the sequences of PbeA and PbeC from *H. jeotgali* A29 as queries for homology searches. In addition to 26 archaeal genomes, 235 additional *pbeAC* genes were identified in diverse bacterial genomes. However, the phylogenetic tree did not result in clear clustering of DndCD into distinct groups according to a proximate DndFGH, PbeAC or neither (Fig. 3). Moreover, even strains within the same species can accommodate different types of Dnd defence systems. For example, *Enterobacter cloacae* e1347 and *E. cloacae* DS15987 harbour PT-based PbeABCD and DndFGH defence systems, respectively, whereas *E. cloacae* B2-DHA possesses only a solitary PT modification system lacking the restriction counterparts, which is consistent with the HGT of Dnd systems (Supplementary Data 3).

Considering the functional association, we next examined whether these PT modification components had coevolved and been horizontally transferred together with PbeABCD, similar to what occurred with DndFGH^34^. Using DndCD and PbeAC of *H. jeotgali* A29 as references, we calculated the alignment similarity rate between the 261 DndCD-PbeAC pairs and their respective reference Dnd and Pbe proteins using the approach previously described^34^. The same calculation was applied to the 1132 coexisting DndCD-DndFGH proteins, with DndFGH of *S. enterica* serovar Cerro 87 used as the reference. In general, the coevolution of the modification and restriction components should lead to a linear relationship between their similarity rates^34^. The results showed that the correlation coefficient (*ρ*) between the similarity rates for DndCD and DndFGH was 0.582, which is consistent with our previous report^34^ and confirms the coevolution of DndCD and DndFGH (Supplementary Fig. 8a). In contrast, a *ρ*-value of 0.107 between DndCD and PbeAC was observed, suggesting that either the *dndCD* and *pbeAC* genes were acquired separately by HGT events or that they evolved at different mutational rates owing to different genetic selection pressures (Supplementary Fig. 8b).

**Fig. 6:**
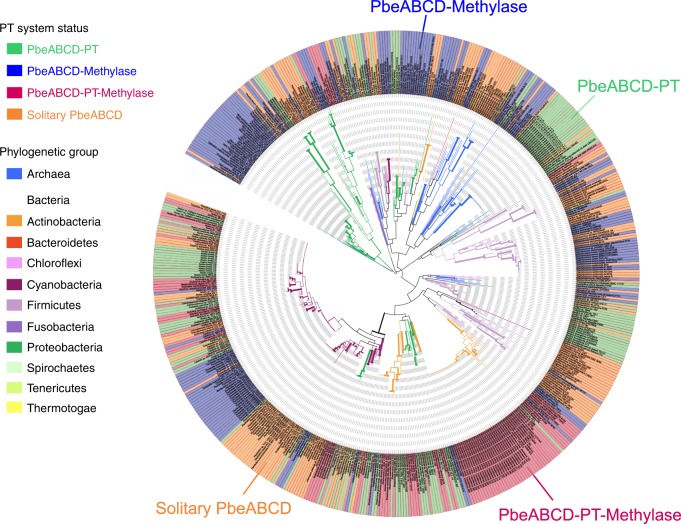
Phylogenetic analysis of 553 PbeAC proteins. Each archaeal and bacterial group is represented in a distinct colour. Green (128) and blue (155) shading indicate the presence of DndCD for phosphorothioate (PT) modification or MTase for methylation within the vicinity of PbeAC. One-hundred and five PbeAC homologues have DndCD and MTase simultaneously present within the defined neighbourhood (1 bp–10 kb), while 165 PbeAC homologues are solitary, lacking the modification component within the neighbourhood. Source data are provided as a Source Data file

To gain a more global view of this new type of PT-based PbeABCD defence system, we performed homology searches and constructed a phylogenetic tree using all 553 PbeAC proteins detected (Fig. 6). An immediate observation was the occurrence of potential interdomain and intradomain gene transfer events. For instance, instead of clustering with other halophilic Archaea, *Halorubrum trapanicum* CBA1232 grouped with a large clade of bacterial strains, including those of the genera *Lactobacillus*, *Staphylococcus* and *Bacillus*. Surprisingly, only 42.1% (233) of 553 PbeAC proteins were observed to be accompanied by *dndCD* within 1 bp–10 kb, raising two possibilities: (1) that the solitary PbeABCD components are not active in host cells because their antiviral activity depends on the presence of PT modification and (2) that PbeABCD has evolved to pair with other DNA modifications, such as methylation, to yield new defensive modules. The latter possibility is supported by the observation that candidate base-modifying proteins are encoded within 10 kb of 155 of 320 solitary PbeAC examples (Supplementary Data 4). Notably, PbeAC and DndFGH were observed to be simultaneously present in the neighbourhood of 25 bacterial *dndCD* clusters (Fig. 3 and Supplementary Data 3). It would be interesting to explore the interactions between these gene cassettes and their contributions to the host defence against invasive DNA.

## Discussion

In this study, we describe a new type of PT-based multi-gene antiviral system in Archaea, DndCDEA-PbeABCD, that is genetically and phenotypically distinct from DndBCDE-DndFGH. In spite of the absence of the transcriptional repressor DndB, the DndCDEA-PbeABCD system in four haloarchaeal strains drives motif-specific DNA PT modifications at frequencies comparable to those observed in Bacteria^32,34^. Approximately half of PT sites occur in the form of hemi-PT-modified 5ʹ-G_PS_ATC-3ʹ/5ʹ-GATC-3ʹ in Archaea, which is in sharp contrast to the predominant full PT modifications that occur in Bacteria^32,34^. However, although PbeABCD allows viral adsorption and DNA injection into the host archaeal cells, it prevents viral DNA replication in a PT-dependent manner. Notably, the DndCDEA-PbeABCD pair was no longer able to protect against a PT-modified SNJ1 virus, suggesting a strategy of utilising epigenetic modification for self/non-self discrimination. We therefore speculated that PbeABCD serves as the REase component to target non-PT-modified viral DNA, analogous to R-M systems. However, two lines of evidence point to a novel mechanism of defence that is different from type II R-M systems, which have autonomous REase and MTase activities, and PT-based DndFGH systems. First, no sign of the processive degradation or cleavage of viral DNA was detected in the presence of DndCDEA-PbeABCD. Second, in typical R-M or DndFGH-mediated Dnd systems, the loss of methylation or PT would be detrimental to cells because the resulting host DNA would be recognised as invading foreign DNA and be attacked by REases or DndFGH. However, *dndCDEA* deletion mutants exhibited no apparent growth defect compared to CJ7-F cells expressing intact *dndCDEA-pbeABCD*, although protection against the virus was lost.

We also showed that all four genes in the *pbeABCD* module appear to be essential for protection against viral infection. PbeA consists of a PLD domain^35^, a Res subunit of Type III REase domain and a helicase domain^39,40^, suggesting that it has multiple enzymatic activities. The PLD domain is present in a wide variety of enzymes involved in phospholipid metabolism, virulence, membrane remodelling and signalling^35^. In addition, it is also recognised as a characteristic of a small family of endonucleases in bacteria, such as Nuc, *Cgl*I, *Ngo*AvII, *Bfi*I and DrmC, a component of the newly identified multi-gene R-M module DISARM^2,41–43^. The Res subunit of Type III REase (i.e., EcoP15I) is a fusion between a DEAD-family helicase, correlating with the DNA translocation activity, and a nuclease resembling the functionally related HsdR subunit of the type I REases^39,40^. Nonetheless, the PD-X-D/EXK motif, the nuclease active site for DNA cleavage, is not present in PbeA, which is consistent with the non-DNA-cleavage defensive activity of PbeABCD. Based on the shared helicase motifs between PbeA and type I and III REases, we, speculate that PbeA might be associated with DNA translocation, looping or sliding to target DNA recognition sites. This possibility would explain why without *pbeA*, the remaining *pbeBCD* still confer a low-level of resistance to SNJ1. While *pbeB* and *pbeD* encode proteins of unknown function sized only 66 and 152 aa, respectively, deletions of them still completely abolished protection against virus SNJ1. Considering their small size, we speculate that PbeB and PbeD might become part of an enzyme complex for stabilisation or functional coupling. Notably, PbeC exhibits sequence resemblance to the structural maintenance of chromosome (SMC) protein (24% identity and 42% similarity) in *Hydrococcus rivularis*. SMC proteins adopt a V-shaped, two-armed architecture with an ATP-binding cassette (ABC)-like domain at the distal end of each arm and are recognised as one of the most fundamental classes of proteins involving in chromosome segregation, recombinational repair and genome-wide gene regulation (Supplementary Data 5)^44^. Although the function and interaction of PbeABCD proteins remain to be further elucidated, these preliminary clues indicate a possible mechanism of action whereby viral DNA molecules are manipulated in a certain manner to inhibit viral replication and propagation. In terms of the PT dependence, we speculate that Pbe protein(s) might sense PT DNA to function or that PbeABCD rely on PT to form an active assembly.

It was interesting to observe the potential HGT events of DndCD between different bacterial phyla, as well as between Archaea and Bacteria. However, based on the phenomenon that DndFGH occurs only in Bacteria and not in Archaea, this interdomain HGT situation does not hold for the restriction component DndFGH. Instead, the PT modifications in Archaea have evolved to be paired with PbeABCD, generating a new type of PT-based defence module with mechanisms of action that differ from those of DndFGH. In addition, PbeABCD-mediated PT defence pairs are also widespread among Bacteria. Further phylogenetic analysis of PbeAC revealed two unusual observations: (1) 320 of 553 *pbeAC* are solitary, lacking the counterpart PT modification *dnd* genes; and (2) *pbeAC*, *dndFGH* and PT modification genes coexist in 25 bacterial strains. Regarding the occurrence of solitary *pbeAC*, it is possible that they are not functional due to their dependence on PT. This scenario is different from that of the REases in type I and III R-M systems, which are activated via protein–protein interactions with the cognate MTases to enable their restriction activity. At present, we still cannot rule out the possibility that PbeABCD has evolved to pair with other modification counterparts, such as MTases, to generate new defensive modules, which is supported by the observation that 155 of 320 solitary *pbeAC* components are in close proximity to genes encoding MTases. In terms of the coexistence of DndFGH, PbeABCD and PT modifications, it is currently unclear whether the first two are both functional or which one pairs with PT or methylation as a defensive module, emphasising the possibility of complicated interactions worthy of future investigation.

In the host–parasite coevolutionary arms race, viruses have developed a variety of counter-resistance tactics to circumvent the arsenal of prokaryotic resistance mechanisms^5^. Considering that the PT-modified SNJ1 virus can bypass PbeABCD-mediated PT defence systems, we can speculate that once the virus completes an infection cycle, the resulting SNJ1 progeny possessing PT modifications would overcome this defence in other archaeal and bacterial cells, regardless of the presence of PbeABCD or DndFGH. Thus, in future studies, it would be interesting to identify viruses with naturally occurring PT modifications or to explore other strategies used by viruses to avoid, circumvent or subvert PT-based defence systems.

Owing to the fairly limited number of archaeal host–virus systems studied, our understanding of archaeal host–virus interactions lags far behind our understanding of those in Bacteria. These limitations also hinder the exploration of new defensive mechanisms in Archaea. In this study, we identified a new type of PT-based Dnd defence system that is widespread in Archaea and Bacteria. The antiviral activity of PbeABCD depends on the presence of DNA PT modification but does not involve viral DNA degradation or cleavage, ruling out the involvement of currently known defence mechanisms. These findings expand our knowledge regarding the arsenal of archaeal defence systems and illuminate the archaeal host–virus arms race.

## Methods

### Strains and growth conditions

All the strains, plasmids and primers used in this study are listed in Supplementary Tables 3 and 4. *Natrinema* sp. CJ7-F and its derivative strains were cultured in Halo-2 or minimal medium (MM, 18%) at 45 °C, as previously described^45^. *H. jeotgali* A29, *H. limi* JCM 16811 and *H. salinum* JCM 19729 were cultivated in JCM medium 402, and *N. bangense* JCM 10635 was grown in JCM medium 167. To construct plasmids, *E. coli* strains were routinely grown in Luria-Bertani (LB) medium at 37 °C. To prepare solid agar medium, agar was added to the medium to a final concentration of 15 g/L. When necessary, ampicillin was added to the medium at a concentration of 100 μg/mL.

### Construction and expression of pWHU3808 and derivatives

The shuttle vector pFJ6-H was used to express *dndCDEA-pbeABCD* from *H. jeotgali* A29 and its derivatives in CJ7-F^45^. Plasmid pWHU3808, expressing the complete *dndCDEA-pbeABCD* module, was constructed in two steps. First, the *dndCDEA* and *pbeABCD* fragments were PCR amplified from *H. jeotgali* A29 genomic DNA using the primer pairs dndCDEA-pYC-F/dndCDEA-pYC-R and pbeABCD-F/pbeABCD-R, respectively. The fragments were inserted into the *Sna*BI-*Hind*III-digested pYCJ-HH and *Not*I-*Sph*I-digested pFJ6-H, yielding pWHU3253 and pWHU3804, respectively. Second, the heat-shock protein 70 (HSP 70) promoter and the *dndCDEA* fusion fragments were PCR amplified from pWHU3253 using the primer pair Hpro-dndCDEA-F/Hpro-dndCEDA-R. The PCR product was then ligated into pWHU3804 digested with *Nde*I and *Kpn*I, yielding pWHU3808. For the in-frame deletion of *pbeA* in pWHU3808, a 5.2 kb fragment containing the downstream portion of *pbeA* was first generated by PCR using the primer pair ΔpbeA-F/ΔpbeA-R from pWHU3808 (Supplementary Table 4). The PCR products and the *Nde*I-*Not*I digested pWHU3808, sharing 25–30 bp identical sequences, were mixed to allow site-specific recombination using a Hieff Clone™ Plus One Step Cloning Kit (Yeasen), generating plasmid pWHU3813. To generate the in-frame deletion of *pbeB* in pWHU3808, the upstream and downstream fragments of *pbeB* were PCR amplified using the primer pairs ΔpbeB-UF/ΔpbeB-UR and ΔpbeB-DF/ΔpbeB-DR, respectively (Supplementary Table 4). The two purified PCR products and the *Nde*I-*Not*I-digested pWHU3808 were mixed according to the manufacturer’s instructions for recombination to generate the plasmid pWHU3814. The procedures used to construct the other pWHU3808-derived plasmids expressing in-frame deletions of the *dnd* genes were similar to those used for pWHU3814. All of the recombinant plasmids were verified by sequencing. The transformation of CJ7-F was performed at room temperature using the polyethylene glycol (PEG) method, as previously described^38^. MM (18%) medium was used to select and propagate CJ7-F transformants.

### Absorption assay

CJ7-F cells expressing or lacking *dndCDEA-pbeABCD* were grown in Halo-2 medium to an OD_600_ of 0.3, followed by infection with virus SNJ1 at a multiplicity of infection (MOI) of 1. During infection, the archaeal cells and virus were incubated with shaking at 45 °C. Aliquots of 1 mL of culture were withdrawn at time points 1, 60, 120, 180, 240 and 300 min post infection, and the cells were pelleted by centrifugation at 10,000 × *g* for 1 min. The unabsorbed SNJ1 concentration in the upper aqueous phase was determined on CJ7-F cell lawns. The percentage of unabsorbed (extracellular) virus SNJ1 was calculated assuming the initial titre of SNJ1 (without added cells) to be 100%.

### Determination of relative SNJ1 levels in CJ7-F hosts

CJ7-F cells harbouring pWHU3808 or the empty vector pFJ6-H, expressing or lacking *dndCDEA-pbeABCD*, respectively, were cultured in 20 mL of Halo-2 medium until the cell density reached an OD600 of 0.3. Logarithmic-phase cultures were infected with the SNJ1 virus at an MOI of 1. Multiple 1-mL samples of the cultures were collected at the indicated time point by centrifugation, and the cells were resuspended in 100 μL of basal salt solution (medium without a carbon source). Total DNA from CJ7-F strains containing the different vectors was prepared as described previously^46^. Briefly, cells resuspended in 100 μL of basal salt solution were lysed after the addition of 900 μL of distilled water. The lysate was diluted 1:100 with distilled water and then was used as the template for real-time qPCR. The levels of the single-copy gene *radA*^38^, located on the chromosome of the host strain, and *repA*, located in the genome of SNJ1, were determined by real-time qPCR to estimate the relative intracellular levels of SNJ1 DNA relative to the CJ7-F chromosome during infection.

### RNA extraction and reverse transcription

Total RNA was extracted from the *H. jeotgali* A29 strain using an Omega RNA Extraction Kit (Omega Bio-Tec) according to the manufacturer’s protocol. Next, the RNA samples were treated with DNase I (Thermo Fisher) to remove residual genomic DNA, and the RNA was then quantified using a NanoDrop2000 spectrophotometer (Thermo Fisher). The RNA was reverse transcribed using a RevertAid First Strand cDNA Synthesis Kit (Fermentas) to obtain cDNA.

### SNJ1 propagation and isolation

The haloarchaeal virus SNJ1 was propagated in *Natrinema* sp. J7-1^45^. The J7-1 strain was grown at 45 °C in Halo-2 medium to late log phase (OD_600_ = 0.8–1.0), after which mitomycin C (Roche) was added to the cultures at a final concentration of 1 μg/mL, followed by overnight incubation at 37 °C with shaking at 200 rpm. The cultures were centrifuged at 10,000 × *g* for 20 min to remove cellular debris, and the supernatants were subsequently filtered through 0.22-µm Millipore pore membrane filters. When necessary, a PEG6000 solution was used to concentrate the phage preparations, as described previously^47^.

### Plaque assays

Halovirus plaque assays were performed as described previously^48^, with slight modifications. Briefly, the CJ7-F derivative strains were grown at 45 °C in 18% MM medium to late log phase (OD_600_ = ~0.8–1.0). Next, an aliquot (400 µL) of the culture was mixed with molten soft agar (0.6%) and poured onto Halo-2 agar plates (1.5%). Once solidified, 5 μL of serial tenfold dilutions of SNJ1 were pipetted onto the agar. The plates were incubated for 36 h at 45 °C, and the phage titres were calculated as PFU (plaque-forming unit)/mL.

### Southern blot analysis

CJ7-F cells possessing pWHU3808 and pFJ6-H were incubated with the SNJ1 virus at an MOI of 5. Multiple aliquots of 10 mL of culture were collected at different time points (1, 5, 10, 15, 30 min and 1, 2, 3, 4, 5 and 6 h) post infection. In addition, samples of the cultures prior to the addition of SNJ1 were used as negative controls. Total genomic DNA was extracted and digested with *Sac*I for Southern blot analysis. The primers SNJ1-southern-F and SNJ1-southern-R were designed to synthesise a probe corresponding to the region from 9243 to 9732 nt of the SNJ1 genome (Supplementary Table 4). The DNA probe preparation, hybridisation and detection were performed using a Detection Starter Kit II (Roche) according to the manufacturer’s instructions.

### Bioinformatic and statistical analysis

Using the DndC, DndD, PbeA and PbeC protein sequences from *H. jeotgali* A29 and the DndF, DndG, and DndH sequences from *S. enterica* serovar Cerro 87 and *Hahella chejuensis* KCTC 2396 as BLAST queries, we searched the NCBI databases of non-redundant nucleotide collections, RefSeq_genomes, WGS, HTGS, refseq_genomic, and GSS. The BLAST hits were filtered using an *e*-value of ≤10^−10^ and an aligned length of ≥30% to the query sequences. The distance between *dndC* and *dndD* was limited to 5 kb or less. Flanking regions of ±20 kb around the *dndCD* cluster were searched for the presence of *pbeA*, *pbeC*, *dndF*, *dndG* and *dndH*. Similarly, we searched for genes encoding MTases or DndCD within close proximity (1 bp–10 kb) to *pbeAC*.

The sequences used to generate the phylogenetic trees were formed by concatenating the protein sequences of DndC and DndD, and PbeA and PbeC. Multiple alignments were performed using MEGA 5.2 with the MUSCLE algorithm. The parameters were set as follows: gap open = −2.9, gap extend = 0, hydrophobicity multiplier = 1.2, max iterations = 8, and min dialogue length = 24, and the UPGMB method was used for clustering. Trees were constructed using the maximum likelihood method with 500 bootstrap replications. The other parameters were set as follows: substitution type = amino acid, substitution model = Jones-Taylor-Thornton model, rates among sites = uniform rates, gaps/missing data treatment = complete deletion, maximum likelihood heuristic method = Nearest-Neighbour-Interchange, and the neighbour-joining method was used to constructed the initial tree. The output trees were visualised by iTOL^49^.

DndCD and PbeAC sequences wereglobally aligned with those of *H. jeotgali* A29 using the needle programme in the EMBOSS package^50^. Similarity values were extracted from the output files. The coevolution analysis has been described in detail previously^34^. Generally, we regressed the similarity values of PbeAC to those of DndCD to explore their coevolution on the basis of the idea that the divergence degree (i.e., 1 minus the similarity rate) of PbeAC and DndCD from their respective ancestor should be linearly related in a given strain, if they evolve at the same speed. The same regression analysis was also performed on DndCD and DndFGH. As *H. jeotgali* A29 lacks *dndFGH*, global alignments were made with the sequence data from *S. enterica* serovar Cerro 87.

All statistical and bioinformatic analyses were performed in R.

## Supplementary information

Supplementary Information

Description of Additional Supplementary Files

Supplementary Data 1

Supplementary Data 2

Supplementary Data 3

Supplementary Data 4

Supplementary Data 5

Reporting summary

Source Data

## Data Availability

The *H. jeotgali* A29, *N. bangense* JCM 10635, *H. salinum* JCM 19729 and *H. limi* JCM 16811 sequencing data generated using the PacBio RSII platform have been deposited in the Sequence Read Archive under the accession numbers SRR7957355, SRR7957354, SRR7957356 and SRR7945240, respectively. The source data for Table 1, Figs. 1 to 6, Supplementary Tables 1 and 2, and Supplementary Figs. 1, 2, 3, 5, 6, and 7 are provided as a Source Data file. The data that support the findings of this study are available from the authors upon request.

## Code availability

Custom codes or software used in this study is available from the authors upon request.
