# Supplementary material for: A new type of DNA phosphorothioation-based antiviral system in archaea
Source: Nat Commun. 2019 Apr 11;10:1688. doi: 10.1038/s41467-019-09390-9 (PMC6459918; doi:10.1038/s41467-019-09390-9)
Supplement: Supplementary file 1 — Supplementary Information [file 41467_2019_9390_MOESM1_ESM.pdf]

Supplementary Information

A new type of DNA phosphorothioation-based antiviral system in archaea

Xiong et al.

Supplementary Table 1. Analysis of consensus sequences in four archaeal strains

H. jeotgali A29	GATC	Downstream base			
		A	C	G	T
Upstream base	A	11.1% (119/1072)	11.0% (159/1445)	2.7% (106/3890)	5.5% (37/670)
	C	5.0% (188/3754)	13.4% (710/5290)	3.2% (343/10834)	3.3% (124/3769)
	G	3.4% (45/1323)	5.0% (78/1556)	**1.2%** (63/5087)	**1.2%** (17/1395)
	T	10.5% (91/870)	**17.8%** (230/1289)	2.6% (97/3798)	3.9% (44/1116)
H. limi JCM 16811	GATC	Downstream base			
		A	C	G	T
Upstream base	A	9.8% (58/593)	9.9% (94/953)	2.7% (50/1856)	9.4% (38/405)
	C	5.6% (102/1820)	10.8% (310/2866)	3.8% (175/4563)	4.7% (86/1830)
	G	2.1% (16/763)	4.2% (51/1205)	**0.6%** (17/2960)	**2.0%** (18/892)
	T	19.1% (81/424)	**22.8%** (172/756)	6.0% (111/1836)	10.5% (63/601)
H. salinum JCM 19729	GATC	Downstream base			
		A	C	G	T
Upstream base	A	1.9% (17/905)	3.2% (1.9/1220)	0.7% (18/2725)	0.9% (5/579)
	C	1.9% (53/2795)	7.4% (273/3678)	1.7% (96/5762)	0.9% (27/2745)
	G	1.2% (12/1009)	4.9% (43/881)	**0.6%** (22/3781)	**0.5%** (6/1135)
	T	8.3% (55/660)	**13.9%** (136/977)	2.6% (71/2739)	4.6% (38/830)
N. bangense JCM 10635	GATC	Downstream base			
		A	C	G	T
Upstream base	A	3.7% (43/1168)	6.5% (81/1241)	1.4% (46/3181)	2.1% (14/656)
	C	4.0% (142/3513)	12.9% (479/3686)	3.5% (270/7691)	2.5% (79/3123)
	G	1.6% (20/1215)	4.8% (42/880)	**0.9%** (31/3567)	**0.6%** (7/1242)
	T	7.2% (73/1013)	**13.4%** (149/1111)	3.1% (107/3467)	2.6% (31/1188)

Data represent the SMRT sequencing-determined frequency of sequences containing the noted bases flanking the 5'-G$_{PS}$ATC-3' consensus sequences in four halophilic archaea. Preferential and rare occurrences of PT modifications were present in the 5'-tGATCc-3' (red) and 5'-gGATC(g/t)-3' (blue) motifs, respectively.

Supplementary Table 2. Analysis of methylation motifs in four archaeal strains by SMRT sequencing

H. jeotgali A29								
Motif	Modified Position	Modification Type	Motifs Detected	# Of Motifs Detected	# Of Motifs In Genome	Mean Modification QV1	Mean Motif Coverage	Partner Motif
CATTC	2	^{m6}A	98.04%	2,554	2,605	127.04	93.66	-
CACCAYG	5	^{m6}A	97.96%	817	834	121.82	97.64	-
GAGGAG	5	^{m6}A	95.87%	5,903	6,157	113.53	96.81	-
CTGACGNNBN NGT	5	^{m4}C	67.56%	152	225	63.61	97.23	-
CTAGBNNGT	1	^{m4}C	40.72%	68	167	56.32	95.22	-
GCAATNNBNN NNNV	4	^{m6}A	22.75%	276	1,213	50.59	104.80	-
GGTRTVGCR	3	unknown	21.08%	78	370	40.10	111.03	-
TCCYAVYW	1	unknown	16.39%	150	915	42.29	117.23	-
GNNNNNNGTH	1	unknown	4.45%	5,674	127,511	37.94	112.01	-
THR	1	unknown	1.10%	6,662	604,625	35.34	139.63	-
***N. bangense* JCM 10635**								
Motif	Modified Position	Modification Type	Motifs Detected	# Of Motifs Detected	# Of Motifs In Genome	Mean Modification QV	Mean Motif Coverage	Partner Motif
CAGATG	4	^{m6}A	99.51%	1,832	1,841	152.16	105.28	-
CATTC	2	^{m6}A	98.74%	4,085	4,137	151.99	102.10	-
CTAGTTCG	1	^{m4}C	81.48%	22	27	82.91	95.32	-
AGGCMGYA	1	^{m6}A	36.87%	153	415	68.86	109.76	-
ANNNACTAG	1	^{m6}A	31.40%	65	207	69.91	100.38	-
TTRBAVBW	1	unknown	16.06%	592	3,687	46.97	107.93	-
TVNNNNNH	1	unknown	3.57%	34,805	973,601	36.79	114.01	-
***H. salinum* JCM 19729**								
Motif	Modified Position	Modification Type	Motifs Detected	# Of Motifs Detected	# Of Motifs In Genome	Mean Modification QV	Mean Motif Coverage	Partner Motif
GAGATC	4	^{m6}A	81.69%	5,096	6,238	69.36	46.61	-
GCATC	3	^{m6}A	73.85%	5,271	7,137	74.21	46.80	-
CTTGAT	5	^{m6}A	73.18%	745	1,018	77.56	47.58	-
CTAGYNNG	1	^{m4}C	52.43%	335	639	52.49	45.52	-
CGATCC	3	^{m6}A	14.20%	1,246	8,777	60.14	34.05	-
HGWNVNVDG	2	unknown	4.55%	4,973	109,331	36.59	49.78	-
***H. limi* JCM 16811**								
Motif	Modified Position	Modification Type	Motifs Detected	# Of Motifs Detected	# Of Motifs In Genome	Mean Modification QV	Mean Motif Coverage	Partner Motif
CGATCC	3	^{m6}A	99.76%	7,133	7,150	149.53	93.40	-
TTCGAA	6	^{m6}A	88.88%	1,303	1,466	109.61	86.55	TTCGAA
RAGGYASYT	2	^{m6}A	43.92%	83	189	59.02	93.49	-
DAGGYVGYA	2	^{m6}A	20.14%	149	740	64.07	92.99	-
TNNNNNNH	1	unknown	2.10%	18,282	869,418	36.65	98.39	-
TVRVNNNG	1	unknown	1.72%	2,692	156,155	35.81	98.57	-

1 QV = quality value

Supplementary Table 3. Strains and plasmids used in this study.

Strains	Characteristics	Reference or Source
E. coli DH5α	*E. coli* host for pFJ6H and its derivatives	Sangon Biotech
Natrinema sp. J7-1	With SNJ1 proviral genome pHH205, cannot be infected by SNJ1	1
Natrinema sp. CJ7-F	Δ*pyrF*, pHH205-free derivative of J7-1, can be infected by SNJ1	2
H. salinum JCM 19729	d(G_{PS}A)	3
H. limi JCM 16811	d(G_{PS}A), d(G_{PS}G)	4
H. jeotgali A29	d(G_{PS}A)	5
N. bangense JCM 10635	d(G_{PS}A)	6
Plasmids	Characteristics	Reference or Source
pYCJ-HH	A shuttle expression plasmid constructed based on the stable replicon of SNJ1	7
pFJ6-H	A shuttle plasmid that can replicates in both *Natrinema* sp. CJ7-F and *E. coli* DH5α	2
pWHU3253	pYCJ-HH derivative harbouring *dndCDEA* from *H. jeotgali* A29	This work
pWHU3803	pFJ6-H derivative for expression of *dndCDEA*	This work
pWHU3804	pFJ6-H derivative for expression of *pbeABCD*	This work
pWHU3789	pFJ6-H derivative for expression of $dndCDEA_{C344S}$-*pbeABCD*	This work
pWHU3808	pFJ6-H derivative for expression of *dndCDEA-pbeABCD*	This work
pWHU3809	pFJ6-H derivative for expression of *ΔdndC-dndDEA-pbeABCD*	This work
pWHU3810	pFJ6-H derivative for expression of *ΔdndD-dndCEA-pbeABCD*	This work
pWHU3811	pFJ6-H derivative for expression of *ΔdndE-dndCDA-pbeABCD*	This work
pWHU3812	pFJ6-H derivative for expression of *ΔdndA-dndCDE-pbeABCD*	This work
pWHU3813	pFJ6-H derivative for expression of *dndCDEA-ΔpbeA-pbeBCD*	This work
pWHU3814	pFJ6-H derivative for expression of *dndCDEA-ΔpbeB-pbeACD*	This work
pWHU3815	pFJ6-H derivative for expression of *dndCDEA-ΔpbeC-pbeABD*	This work
pWHU3816	pFJ6-H derivative for expression of *dndCDEA-ΔpbeD-pbeABC*	This work

Supplementary Table 4. Primers used in this study

Primers	Sequence (5'-3')	Reference or Source
Heterologous expression		
dndCDEA-pYC-F	TACGTAACGGTTTCACAATCT	This study
dndCDEA-pYC-R	AAGCTTAAGTCGCTGAGTCTAG	This study
dndCDEA-pFJ-F	TATTTGCGGGCAAGGGCGGCCGCACGGTTTCACAATCT	This study
dndCDEA-pFJ-R	GATTACGCCAAGCTTGCATGCAAGTCGCTGAGTCTAG	This study
pbeABCD-F	TATTTGCGGGCAAGGGCGGCCGCATGCCTGAGGACAAGCCGAATC	This study
pbeABCD-R	GATTACGCCAAGCTTGCATGCGAGTTGCTGTCGTAGCGAAACG	This study
Hpro-dndCDEA-F	AGAGCAGATTGTACTGAGAGTGCACCATATGCGCGTCGACCGCCCTCGAGGCGAGC	This study
Hpro-dndCDEA-R	AAGAATAAGCCGAGATGGATCCCCGGGTACCCAGCTATGACCATGATTACGCCTCA	This study
In-frame gene deletion		
ΔdndC-3F	GTTGAGCTTCATTTCTCGATCCACTCCCTATAAGATTGTG	This study
ΔdndC-2R	CACAATCTTATAGGGAGTGGATCGAGAAATGAAGCTCAAC	This study
ΔdndD-3F	GGTTGAGGTCTTTACTCATTTTCTCGATTTACTCCATCAT	This study
ΔdndD-2R	ATGATGGAGTAAATCGAGAAAATGAGTAAAGACCTCAACC	This study
ΔdndE-2F	GAGTCTGTGGTCATGATTCTAGGTCTTTACTCATTGTTGA	This study
ΔdndE-2R	TCAACAATGAGTAAAGACCTAGAATCATGACCACAGACTC	This study
ΔdndA-F	AGAGCAGATTGTACTGAGAGTGCACCATATGCGCGTCGACCGCCCTCGAGGCGAGC	This study
ΔdndA-R	TAAGCCGAGATGGATCCCCGGGTACCGACTTCCCGATCAGAGTCTG	This study
ΔpbeA-F	AAGACGGTATTTGCGGGCAAGGGCGGCCGCGTCTGTAAGAAGCAGAGCAG	This study
ΔpbeA-R	CGACGGGCTCGCCTCGAGGGCGGTCATATGGTGCACTCTCAGTACAATCT	This study
ΔpbeB-UF	ATGAATCAGAGCTATTAGCGGTACTTAATGAGGATTACACGAATTTCACT	This study
ΔpbeB-UR	CGGTATTTGCGGGCAAGGGCGGCCGCATGCCTGAGGACAAGCCGAATCCA	This study
ΔpbeB-DF	ACGGGCTCGCCTCGAGGGCGGTCATATGGTGCACTCTCAGTACAATCTGC	This study
ΔpbeB-DR	TTATATCGGAGGGGTTGGTCAAGAGCATGCCAAGCTTGGCGTAATCATG	This study
ΔpbeC-UF	TATTTGCGGGCAAGGGCGGCCGCATGCCTGAGGACAAG	This study
ΔpbeC-UR	ACGACACAGGAAAGACATAATGGCTCGAACGCTCA	This study
ΔpbeC-DF	TGAGCGTTCGAGCCATTATGTCTTTCCTGTGTCGT	This study
ΔpbeC-DR	CGCCTCGAGGGCGGTCATATGGTGCACTCTCAGTAC	This study
ΔpbeD-UF	TATTTGCGGGCAAGGGCGGCCGCATGCCTGAGGACAAG	This study
ΔpbeD-UR	ATGCTTACGTTCGAGCCATTAGTCATCA	This study
ΔpbeD-DF	GAACGTAAGCATGCCAAGCTTG	This study
ΔpbeD-DR	CGCCTCGAGGGCGGTCATATGGTGCACTCTCAGTAC	This study
Point mutation		
$dndA_{C344S}$-UF	GTACTGAGAGTGCACCATATGACCGCCCTCGAGGCG	This study
$dndA_{C344S}$-UR	CACTCGCACTAGCCGAC	This study
$dndA_{C344S}$-DF	GTCGGCTAGTGCGAGTG	This study
$dndA_{C344S}$-DR	CGAGATGGATCCCCGGGTACCCAGCTATGACCATGA	This study
Real-time qPCR assay		
radA-F	CGTCAACGTCCAGCTTCCACAG	2
radA-R	GAGCCTTCGATTTCGCGGTCC	2
SNJ1-F	GCGGAAATACCCGAACACCAAG	2
SNJ1-R	CTCGCCACAGCAGTCGCAGAT	2
Southern blot assay		
SNJ1-southern-F	AAGATGAGATCCGAGGGAAGTGGCT	This study
SNJ1-southern-R	CGCTGCGTCTCGCTCCAAACT	This study
RT-PCR		
Primer A	ATGATCTGTTGGTTCTCCCTG	This study
Primer B	CTCTATGGCATCTCGGACCT	This study
Primer C	GGCTAAGGATCACGGAGAAC	This study
Primer D	CGTACTTCAGCACTTCCTTCC	This study
pbeC-D-F	TATCGCCTGAGTCTATTG	This study
pbeC-D-R	TTCATCTACTGCCTTCTG	This study

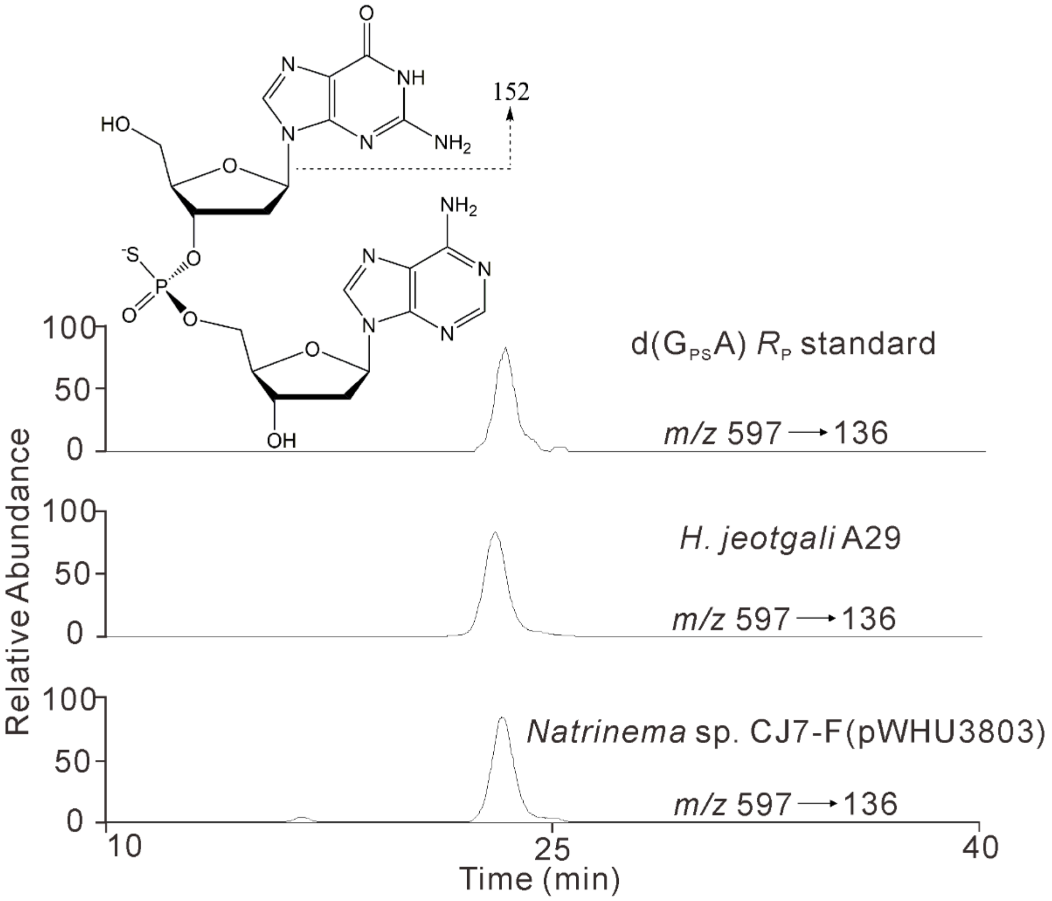

Supplementary Figure 1. The PT modifications in archaeal strains detected using liquid chromatography-coupled tandem quadrupole mass spectrometry (LC-MS/MS). Naturally occurring d(G_{PS}A), with the protonated molecular ion [M+H] appearing at *m/z* 597, in *H. jeotgali* A29 exhibited a retention time identical to that of synthetic d(G_{PS}A) R_P. Plasmid pWHU3803, expressing the *dndCDEA* operon from *H. jeotgali* A29, conferred d(G_{PS}A) in *Natrinema* sp. CJ7-F. The fragmentation pattern of d(G_{PS}A) is shown in the structural inset. Source data are provided as a Source Data file.

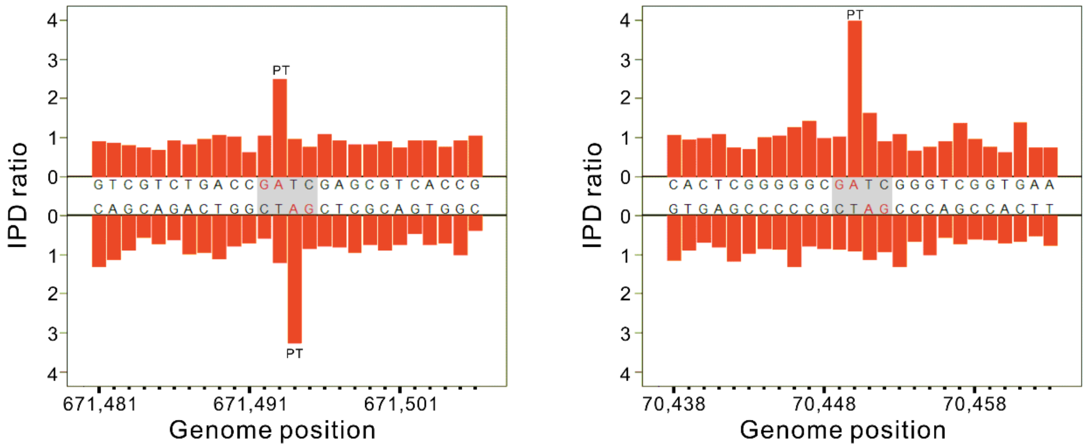

Supplementary Figure 2. SMRT sequencing analysis reveals full and hemi-PT modifications in archaeal genomes. Example IPD (interpulse duration) ratio plots showing instances of fully and hemi-PT-modified 5’-$\mathrm{G_{PS}}$ATC-3’/5’-$\mathrm{G_{PS}}$ATC-3’ and 5’-$\mathrm{G_{PS}}$ATC-3’/5’-GATC-3’, respectively, in *H. jeotgali* A29. Source data are provided as a Source Data file.

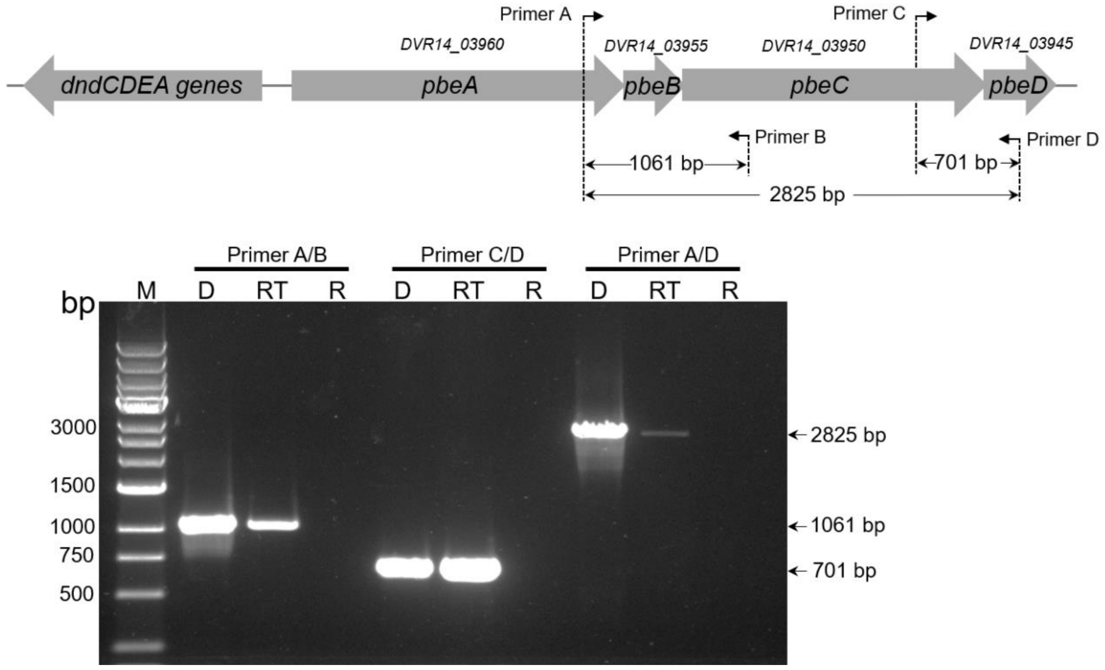

Supplementary Figure 3. RT-PCR analysis of the co-transcription of *DVR14_03960-DVR14_03955-DVR14_03950-DVR14_03945* (*pbeABCD*). The primers are schematically located above or below the genes. The PCR products were obtained using genomic DNA (lanes marked with "D"), reverse-transcribed cDNA (lanes marked with "RT") and untranscribed RNA (lanes marked with "R") from *H. jeotgali* A29 as the template. Primers A and B were used to produce the 1,061-bp PCR fragment; primers C and D were used to generate the 701-bp PCR fragment; and primers A and D were used to produce the 2,825-bp PCR fragment. Source data are provided as a Source Data file.

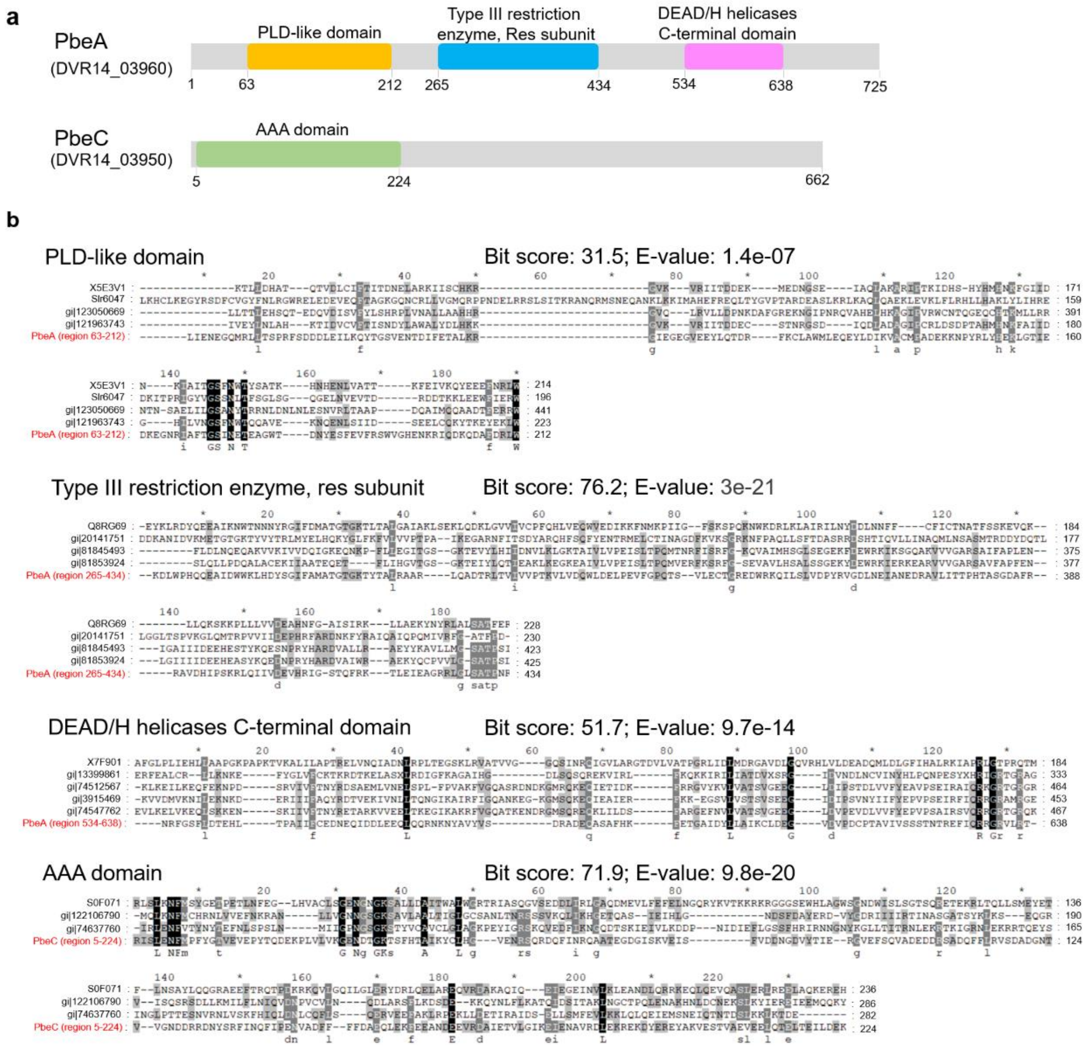

Supplementary Figure 4. Pbe proteins, their domain annotations and sequence alignments. **a** Domain annotations of Pbe proteins using the Pfam version 32.0 database. The domain annotation scores and e-value are listed in Supplementary Data 5. **b** Sequence alignments of individual domain of PbeA and PbeC with homologous domains. The domain-based multiple sequence alignments of these candidate domains with other similar domain architectures were performed using ClustalW in MEGA software version 7.

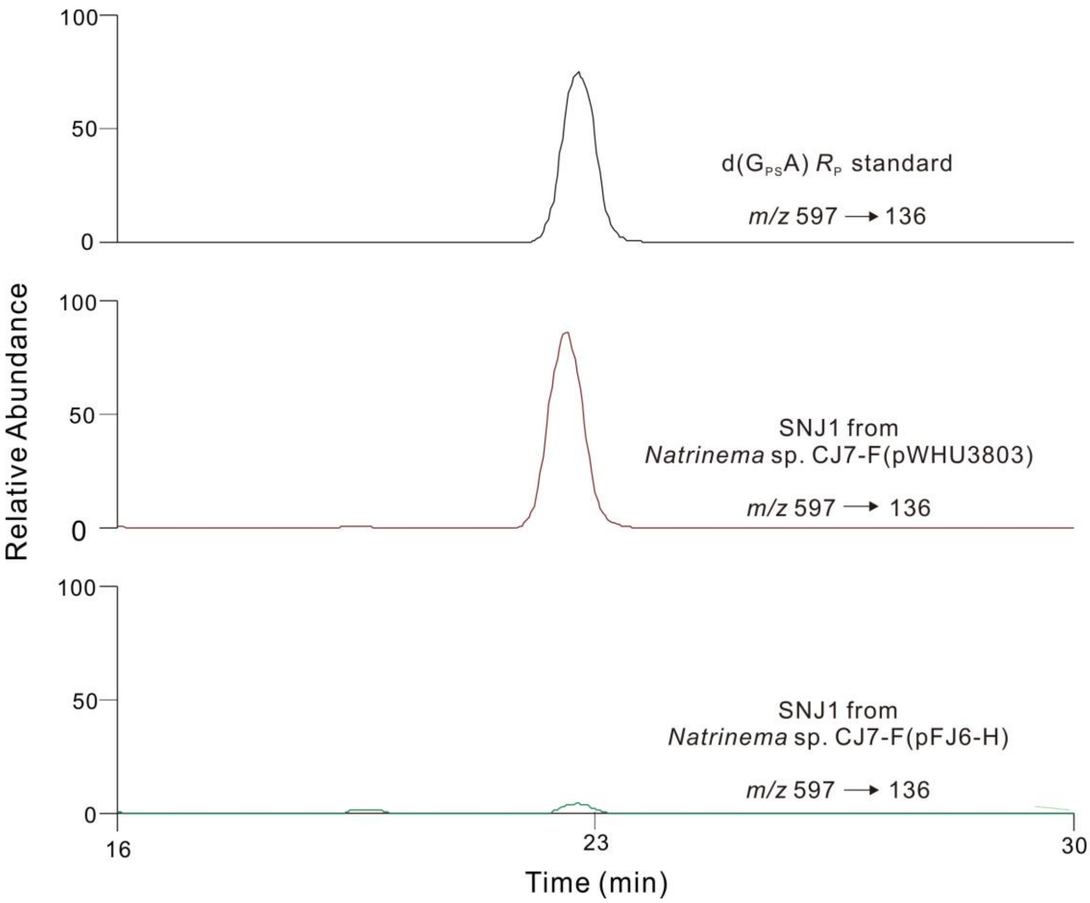

Supplementary Figure 5. LC-MS/MS analysis of PT modification in the SNJ1 virus. PT-modified d(G_{PS}A) was detected in the SNJ1 virus after propagation in CJ7-F(pWHU3803) cells expressing *dndCDEA* from *H. jeotgali* A29. No PT modification was detected when the SNJ1 virus was propagated in CJ7-F cells in the presence of the empty plasmid pFJ6-H. Source data are provided as a Source Data file.

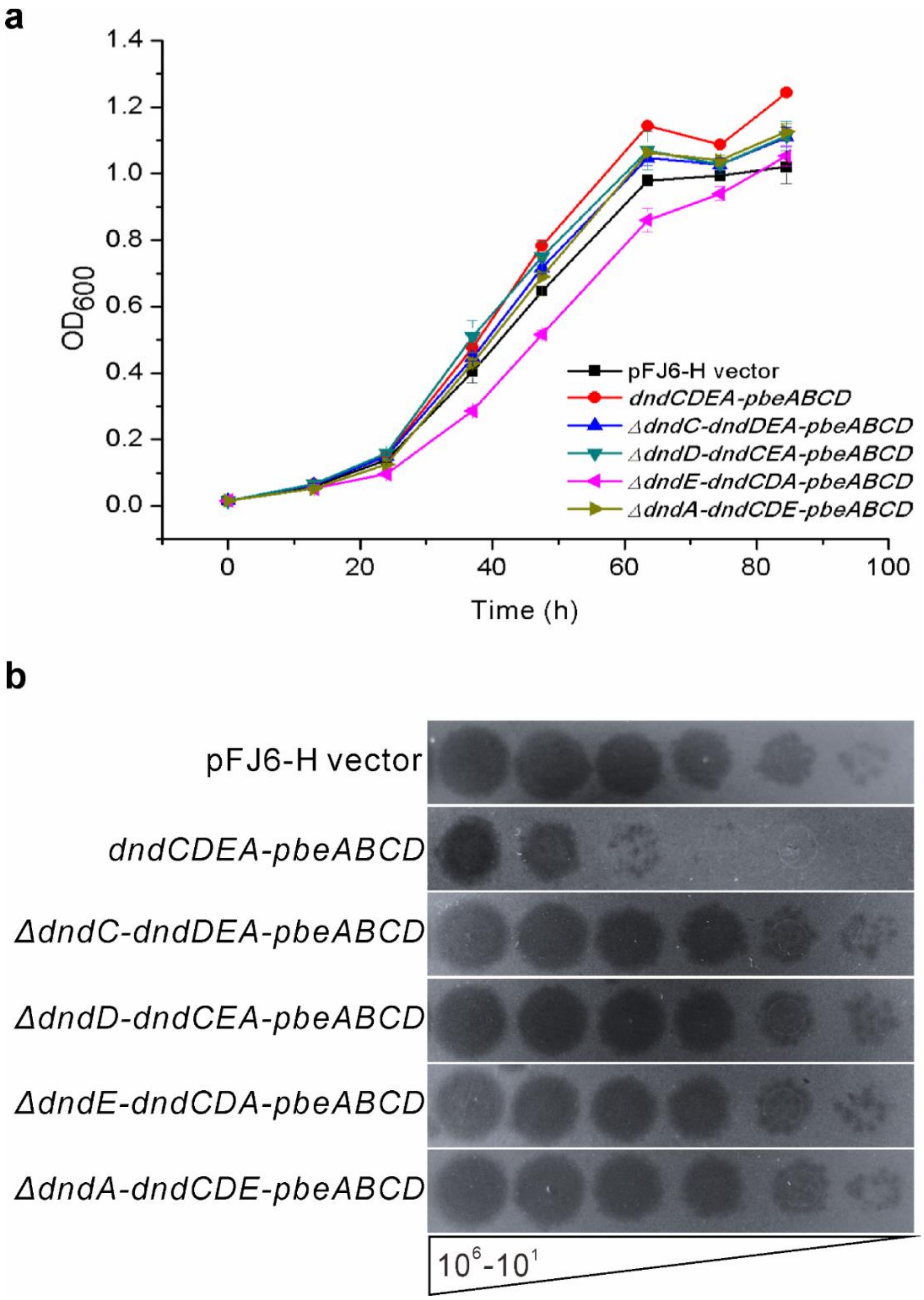

Supplementary Figure 6. Growth profiles and phage resistance of CJ7-F cells expressing DndCDEA-PbeABCD and variants. **a** CJ7-F cells carrying pWHU3809, pWHU3810, pWHU3811 or pWHU3812, expressing the inactivated *dndCDEA* genes but the complete *pbeABCD* cluster, displayed similar growth profiles to that of CJ7-F cells containing the empty vector pFJ6-H. **b** The individual deletion of each of the *dndCDEA* genes restored the sensitivity of CJ7-F cells to the SNJ1 virus. Source data are provided as a Source Data file.

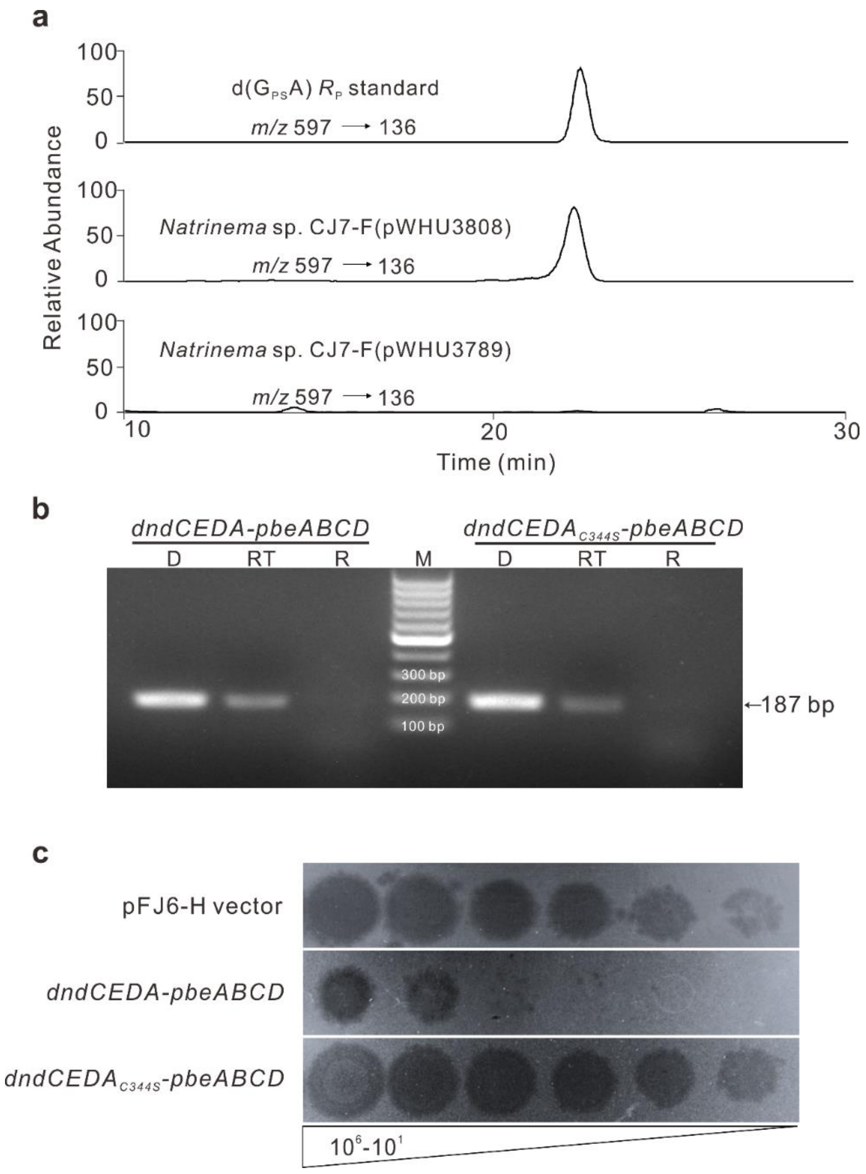

Supplementary Figure 7. The effects of a single point mutation in DndA on DNA PT modification, *pbe* module transcription and antiviral activity. **a** The single point mutation C344S in DndA in pWHU3789 resulted in the loss of the DNA PT modification in d(G_{PS}A). This mutation had no effect on the transcription of the *pbe* operon (**b**) but caused failure to reduce the ability of the SNJ1 virus to form plaques (**c**). PCR products were obtained using genomic DNA (lanes marked with “D”), reverse-transcribed cDNA (lanes marked with “RT”) and untranscribed RNA (lanes marked with “R”) from CJ7-F(pWHU3808) and CJ7-F(pWHU3789) as templates, respectively. Primers pbeC-D-F and pbeC-D-R were listed in Supplementary Table 4. Source data are provided as a Source Data file.

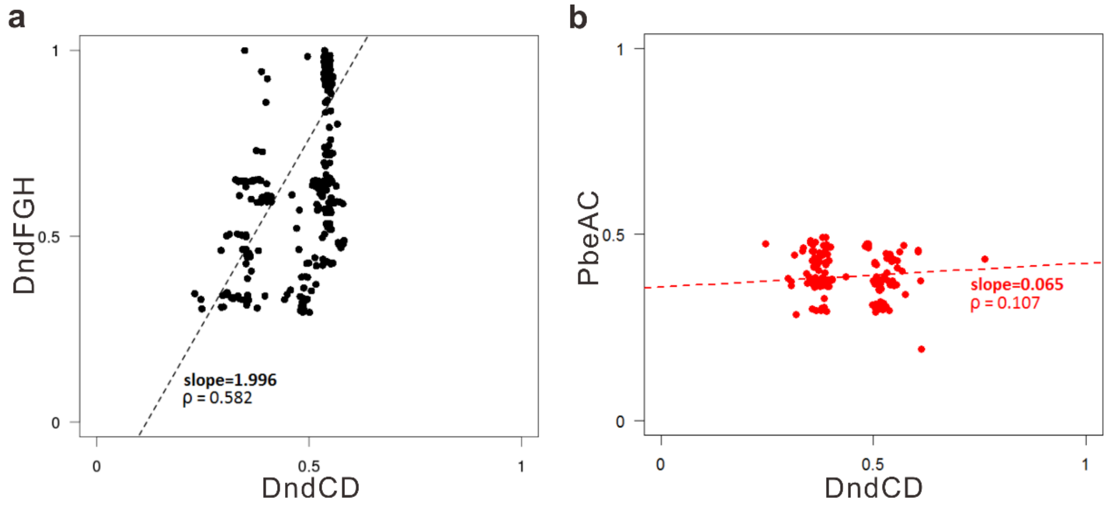

Supplementary Figure 8. Co-evolutionary comparison of the DndCD-PbeAC and DndCD-DndFGH systems. For strains harbouring both *pbeAC* and *dndCD*, we aligned the PbeAC and DndCD protein sequences with those of *H. jeotgali* A29. The alignment similarity rates of the former were regressed on the alignment similarity rates of the latter. A similar procedure was applied to the strains with both *dndFGH* and *dndCD*. Since *H. jeotgali* A29 lacks *dndFGH*, alignments were made with the sequence data from *S. enterica* serovar Cerro 87. **a** The high correlation coefficient (ρ = 0.582) suggests the co-evolution of the DndCD and DndFGH components, consistent with previous observations. **b** In contrast, the low correlation coefficient (ρ = 0.107) indicates a separate evolutionary process for DndCD and PbeAC.

References

1. Zhang, Z. et al. Temperate membrane-containing halophilic archaeal virus SNJ1 has a circular dsDNA genome identical to that of plasmid pHH205. *Virology* **434**, 233-241 (2012).
2. Wang, Y., Chen, B., Sima, L., Cao, M. & Chen, X.J.A. Construction of expression shuttle vectors for the Haloarchaeon natrinema sp. J7 based on its chromosomal origins of replication. *Archaea* **2017**(2017).
3. Song, H.S. et al. Halapricum salinum gen. nov., sp. nov., an extremely halophilic archaeon isolated from non-purified solar salt. *Antonie van Leeuwenhoek* **105**, 979-986 (2014).
4. Cui, H. et al. Halobellus limi sp nov and Halobellus salinus sp nov., isolated from two marine solar salterns. *International Journal of Systematic and Evolutionary Microbiology* **62**, 1307-1313 (2012).
5. Cha, I.-T. et al. Genome sequence of the haloarchaeon Haloterrigena jeotgali type strain A29T isolated from salt-fermented food. *Standards in Genomic Sciences* **10**, 49 (2015).
6. Xu, Y., Zhou, P. & Tian, X. Characterization of two novel haloalkaliphilic archaea Natronorubrum bangense gen. nov., sp. nov. and Natronorubrum tibetense gen. nov., sp. nov. *International Journal of Systematic and Evolutionary Microbiology* **49**, 261-266 (1999).
7. Wang, Y. et al. Identification, characterization, and application of the replicon region of the halophilic temperate sphaerolipovirus SNJ1. *Journal of bacteriology* **198**, 1952-1964 (2016).
