# Supplementary material for: A new type of DNA phosphorothioation-based antiviral system in archaea
Source: Nat Commun. 2019 Apr 11;10:1688. doi: 10.1038/s41467-019-09390-9 (PMC6459918; doi:10.1038/s41467-019-09390-9)
Supplement: Supplementary file 2 — Description of Additional Supplementary Files [file 41467_2019_9390_MOESM2_ESM.docx]

**Description of Supplementary Files**

**File Name:** Supplementary Data 1.

**Description:** 2642 strains with dndCD homologs

**File Name:** Supplementary Data 2.

**Description:** PT distribution in four archaeal strains

**File Name:** Supplementary Data 3.

**Description:** 2361 dndCD and surrounding PbeAC and DndFGH

**File Name:** Supplementary Data 4.

**Description:** 553 PbeAC and surrounding DndCD and MTase

**File Name:** Supplementary Data 5.

**Description:** homologs of DVR14_03960 and DVR14_03950 and domain architecture
