# Supplementary material for: A new type of DNA phosphorothioation-based antiviral system in archaea
Source: Nat Commun. 2019 Apr 11;10:1688. doi: 10.1038/s41467-019-09390-9 (PMC6459918; doi:10.1038/s41467-019-09390-9)
Supplement: Supplementary file 8 — Reporting summary [file 41467_2019_9390_MOESM8_ESM.pdf]

nature research

Corresponding author(s): DBPR, NCOMMS-18-34310B

Last updated by author(s): Mar 6, 2019

Reporting Summary

Nature Research wishes to improve the reproducibility of the work that we publish. This form provides structure for consistency and transparency in reporting. For further information on Nature Research policies, see Authors & Referees and the Editorial Policy Checklist.

Statistics

For all statistical analyses, confirm that the following items are present in the figure legend, table legend, main text, or Methods section.

n/a	Confirmed	
☐	☒	The exact sample size (*n*) for each experimental group/condition, given as a discrete number and unit of measurement
☐	☒	A statement on whether measurements were taken from distinct samples or whether the same sample was measured repeatedly
☒	☐	The statistical test(s) used AND whether they are one- or two-sided<br>*Only common tests should be described solely by name; describe more complex techniques in the Methods section.*
☒	☐	A description of all covariates tested
☒	☐	A description of any assumptions or corrections, such as tests of normality and adjustment for multiple comparisons
☐	☒	A full description of the statistical parameters including central tendency (e.g. means) or other basic estimates (e.g. regression coefficient) AND variation (e.g. standard deviation) or associated estimates of uncertainty (e.g. confidence intervals)
☐	☒	For null hypothesis testing, the test statistic (e.g. *F*, *t*, *r*) with confidence intervals, effect sizes, degrees of freedom and *P* value noted<br>*Give P values as exact values whenever suitable.*
☒	☐	For Bayesian analysis, information on the choice of priors and Markov chain Monte Carlo settings
☒	☐	For hierarchical and complex designs, identification of the appropriate level for tests and full reporting of outcomes
☒	☐	Estimates of effect sizes (e.g. Cohen's *d*, Pearson's *r*), indicating how they were calculated

Our web collection on statistics for biologists contains articles on many of the points above.

Software and code

Policy information about availability of computer code

Data collection

Both online and local NCBI BLAST (v2.2.30+) were used to search the NCBI database. The license of NCBI BLAST: Public domain

Data analysis

Custom R (v3.5.1) codes and EMBOSS packages (v6.4.0) were used to analyze the search results. Multiple alignments and phylogenetic tree constructions were performed using MEGA (v5.22). Trees were visualized by iTOL (v4.3).

The licenses for the above software were described as follow:
R: GNU GPL
EMBOSS: GNU GPL and LGPL
MEGA: protected under the copyright law, but FREE for use in research and education. It is not allowed to redistribute the MEGA software and associated materials partially or fully in any form.
iTOL: freely available and open to everyone

For manuscripts utilizing custom algorithms or software that are central to the research but not yet described in published literature, software must be made available to editors/reviewers. We strongly encourage code deposition in a community repository (e.g. GitHub). See the Nature Research guidelines for submitting code & software for further information.

Data

Policy information about availability of data

All manuscripts must include a data availability statement. This statement should provide the following information, where applicable:

- Accession codes, unique identifiers, or web links for publicly available datasets
- A list of figures that have associated raw data
- A description of any restrictions on data availability

The H. jeotgali A29, N. bangense JCM 10635, H. salinum JCM 19729 and H. limi JCM 16811 sequencing data generated using the PacBio RSII platform have been deposited in the Sequence Read Archive under the accession numbers SRR7957355, SRR7957354, SRR7957356 and SRR7945240, respectively.

The source data underlying Table 1, Fig. 1 to 6 and Supplementary Table 1, 2, Supplementary Figure 1 to 8 are provided as a Source Data file.

Field-specific reporting

Please select the one below that is the best fit for your research. If you are not sure, read the appropriate sections before making your selection.

☒ Life sciences ☐ Behavioural & social sciences ☐ Ecological, evolutionary & environmental sciences

For a reference copy of the document with all sections, see nature.com/documents/nr-reporting-summary-flat.pdf

Life sciences study design

All studies must disclose on these points even when the disclosure is negative.

Sample size	No sample-size caculation was performed.
Data exclusions	No data excusion.
Replication	Values represent the mean ± SD of three analyses.
Randomization	This is no revevant to our study. This study focuses on the interaction of archaeal hosts and viruses.
Blinding	This is no revevant to our study. This study focuses on the interaction of archaeal hosts and viruses.

Reporting for specific materials, systems and methods

We require information from authors about some types of materials, experimental systems and methods used in many studies. Here, indicate whether each material, system or method listed is relevant to your study. If you are not sure if a list item applies to your research, read the appropriate section before selecting a response.

Materials & experimental systems

n/a	Involved in the study	
☒	☐	Antibodies
☒	☐	Eukaryotic cell lines
☒	☐	Palaeontology
☒	☐	Animals and other organisms
☒	☐	Human research participants
☒	☐	Clinical data

Methods

n/a	Involved in the study	
☒	☐	ChIP-seq
☒	☐	Flow cytometry
☒	☐	MRI-based neuroimaging
